# The chromatin accessibility and transcriptomic landscape of the aging mice cochlea and the identification of potential functional super-enhancers in age-related hearing loss

**DOI:** 10.1186/s13148-024-01702-1

**Published:** 2024-07-04

**Authors:** Chanyuan Zhang, Ting Yang, Xiaoqin Luo, Xiaoqing Zhou, Menglong Feng, Wei Yuan

**Affiliations:** 1 Chongqing Medical University, No. 1 Medical College Road, Yuzhong District, Chongqing, 400016 China; 2 Department of Otolaryngology and Head and Neck, Chongqing General Hospital, Chongqing, 401147 China; 3 Hospital of Traditional Chinese Medicine Affiliated to Southwest Medical University, Luzhou, 646099 China

**Keywords:** Age-related hearing loss, Chromatin accessibility, Epigenetics, Senescence, Super-enhancer

## Abstract

**Background:**

Presbycusis, also referred to as age-related hearing loss (ARHL), is a condition that results from the cumulative effects of aging on an individual's auditory capabilities. Given the limited understanding of epigenetic mechanisms in ARHL, our research focuses on alterations in chromatin-accessible regions.

**Methods:**

We employed assay for transposase-accessible chromatin with high-throughput sequencing (ATAC-seq) in conjunction with unique identifier (UID) mRNA-seq between young and aging cochleae, and conducted integrated analysis as well as motif/TF-gene prediction. Additionally, the essential role of super-enhancers (SEs) in the development of ARHL was identified by comparative analysis to previous research. Meanwhile, an ARHL mouse model and an aging mimic hair cell (HC) model were established with a comprehensive identification of senescence phenotypes to access the role of SEs in ARHL progression.

**Results:**

The control cochlear tissue exhibited greater chromatin accessibility than cochlear tissue affected by ARHL. Furthermore, the levels of histone 3 lysine 27 acetylation were significantly depressed in both aging cochlea and aging mimic HEI-OC1 cells, highlighting the essential role of SEs in the development of ARHL. The potential senescence-associated super-enhancers (SASEs) of ARHL were identified, most of which exhibited decreased chromatin accessibility. The majority of genes related to the SASEs showed obvious decreases in mRNA expression level in aging HCs and was noticeably altered following treatment with JQ1 (a commonly used SE inhibitor).

**Conclusion:**

The chromatin accessibility in control cochlear tissue was higher than that in cochlear tissue affected by ARHL. Potential SEs involved in ARHL were identified, which might provide a basis for future therapeutics targeting SASEs related to ARHL.

**Supplementary Information:**

The online version contains supplementary material available at 10.1186/s13148-024-01702-1.

## Introduction

Presbycusis, or age-related hearing loss (ARHL), is the gradual deterioration of hearing ability associated with aging and is becoming more common as the population ages [1]. According to the World Health Organization, there will be more than 500 million individuals who will suffer significant impairment from presbycusis as the number of people over 60 years of age reaches 1.2 billion worldwide [2]. ARHL is characterized by a symmetrical, progressive decline in hearing function and can have various negative impacts on daily life, including communication difficulties, depression, and even dementia[3].

Age-related diseases can be understood as a form of accelerated aging that occurs as a result of interactions between an individual’s genetic background and various environmental and lifestyle factors throughout their lifetime [4]. ARHL can be attributed to a combination of intrinsic factors, including genetic and epigenetic predisposition factors, and extrinsic factors such as noise and ototoxic medications [5]. Epigenetic factors, such as histone acetylation and DNA methylation, which can influence how genes are expressed without altering the underlying DNA sequence, may also play crucial roles in the variability of hearing loss among older adults [6–8]. Histone acetylation is one of the best-characterized chromatin modifications, and it is generally associated with an open chromatin structure and active transcription [9]. The dynamic nature of these epigenetic modifications enables cellular plasticity and adaptation to different environmental cues. However, how chromatin accessibility changes and shapes transcriptome patterns during ARHL occurrence and subsequent progression remains poorly understood.

Enhancers, or cis-regulatory elements (CREs), play crucial roles in controlling the spatial and temporal expression of genes and are essential for various biological processes [10, 11]. Luo et al. identified and validated two previously unknown Atoh1 enhancers and revealed that the cooperative action of three distinct enhancers underpins effective Atoh1 regulation during hair cell (HC) development [12]. Emerging research has provided evidence that certain regions of animal genomes can accumulate groups of enhancers with exceptionally strong enrichment for the binding of transcriptional coactivators, forming what is known as super-enhancers (SEs) [13]. SEs differ from typical enhancers (TEs) in that they exhibit high levels of histone H3 lysine 27 acetylation (H3K27ac) density and are characterized by their enormous size [14]. Ines Sturmlechner et al. identified 40 senescence-associated super-enhancers (SASEs) of mouse embryonic fibroblasts (MEFs) that are present regardless of how the cells became senescent, with 50 activated genes located near these enhancers [15]. By using gene knockdown techniques and analyzing the core biological properties of senescent cells, researchers have determined that a relatively large number of these SE-regulated genes are involved in supporting the survival of senescent MEFs [16]. This suggests that certain genetic programs activated by SEs can promote the persistence or viability of senescent cells. However, SEs are cell type specific, and there is limited research on how chromatin accessibility changes in ARHL mouse cochleae following senescence.

The formation and maintenance of SEs are also closely linked to chromatin accessibility [17]. Therefore, the identification and mapping of accessible chromatin regions (ACRs) are crucial for deciphering the complex transcriptional regulatory networks underlying gene expression in ARHL mice. Understanding how chromatin accessibility is impacted by epigenetic factors can provide insights into the regulatory consequences of senescence events in ARHL. To assess the chromatin accessibility, this study used high-throughput ATAC-seq, which has been successfully applied to identify alterations in chromatin accessibility in genomic studies of animals and cells, to uncover the transcriptional regulatory networks reorganization of the 3D chromatin architecture of rice genomes during heat stress [18, 19].

In this study, a genome-wide gene difference analysis of transcriptome and chromatin accessibility was conducted between young and aging cochleae, aiming to compare the gene expression and chromatin openness patterns between the two groups. The combined analysis of potential regulatory factors, motifs, and peak numbers in the promoter contributed to a focus on the identification of candidate SEs that might create age-related changes in the cochlear transcriptome. The investigation of chromatin accessibility in relation to SASEs provided valuable insights into the regulatory mechanisms of ARHL underlying cell-specific gene expression and cell identity. In addition, to elaborate on the role of SASEs and SASE genes in the process of HC degeneration in ARHL, we exploited the D-gal (D-Galactose)-induced aging cell model as previously reported for further verification [20, 21]. We revealed that the expression of the majority of SASE genes changed significantly after treatment with D-gal, and the inhibition of SASEs induced by JQ-1 (a small molecule inhibitor that has been widely used in research to disrupt the activity of SEs [22]) affected the mRNA levels of SASE genes. Thus, by considering both chromatin accessibility and gene expression data, we gained a more comprehensive understanding of the regulatory mechanisms involved in age-related changes in the cochlea; the regulation of SASEs and SASE genes might be a new therapeutic strategy for the prevention and treatment of ARHL.

## Material and methods

### Animals and model establishment

C57BL/6J mice were divided into two groups: control:6W (6 week old, n = 12) and aging:12 M (12 month old, n = 17), with gender not restricted. The experiments were performed in strict accordance with the Guide for the Care and Use of Laboratory Animals (Ministry of Science and Technology of the Peoples Republic of China, Policy No. 2006398). Experiments involving animals had approval from the Chongqing Medical University Animal Welfare Committee.

### Auditory brainstem response (ABR) measurements

The ABR test was performed using the TDT BioRigRZ system (Tucker-Davis Technologies, USA), as directed by the manufacturer. After the application of anesthesia, subcutaneous needle-like electrodes were inserted under the mice skin at specific locations. The mice underwent anesthesia kept warm in the course of ABR recordings. To evoke auditory brainstem response (ABR) potentials, tone pips with a duration of 5 ms were administered to the eardrum at different frequencies (4 kHz, 8 kHz, 16 kHz, 24 kHz, and 32 kHz). The minimal stimulus levels producing reproducible responses for ABR wave II in various animals were determined as the ABR thresholds. All above frequencies were measured.

### Tissue preparation and hematoxylin and eosin (H&E) staining

The freshly removed cochleae were placed into glass vials with fresh 4% PFA (Thermo scientific, Rockford, IL) overnight at 4 °C after washed by precooled phosphate-buffered saline (PBS, 0.1 M, pH 7.6). After fixation, the cochleae were washed for 3 × 10 min in PBS and then decalcified in 10% EDTA (ethylenediamine tetra-acetate, Sigma) in PBS at 4 °C. The cochleae samples were checked daily until decalcification was complete (approximately 2–3 days). Then the cochleae were washed 3 × 10 min in PBS and then dehydrated with 10%, 20%, and 30% sucrose solution at 4 °C for 1 h, 2 h, and overnight, respectively, until the cochleae sank. The next day, the dehydrated cochleae were infiltrated and embedded in a molten paraffin wax and cut to 5 μm. Sections of one cochlea per mouse were staining with hematoxylin and eosin for histopathology to identify histopathological details for stria vascularis (SV) thickness from the portions of the basal, middle, and apical turns. The thickness of SV was determined by averaging three distance measurements taken perpendicular to the surface of strial marginal cells.

### TUNEL assay

H&E staining sections for SV thickness measurement were also examined for spiral ganglion cells (SGCs) using light microscopy. To evaluate SGCs apoptosis, adjacent serial slides were tested by the TUNEL (terminal deoxynucleotidyl transferase-mediated dUTP nick-end labeling) assay kit (Beyotime, Shanghai, China) as directed by the manufacturer. In brief, cochlear specimens were washed for 3 × 10 min in PBST and underwent staining with TUNEL working solution at 37 °C for 1 h away from light. Nuclei were stained with DAPI and photographed using an Olympus BX63 microscope.

### Whole-mount staining

Cochlear specimens underwent fixation at 4 °C with 4% PFA overnight and decalcification with 10%EDTA for 72 h at 4 °C. Cochleae were exposed to the sensory epithelium and divided into basal, middle, and apical segments. They were blocked for 30 min with blocking reagent (10% normal goat serum in 0.01 M PBS), followed by overnight incubation with primary antibodies, anti-myosin7a (1:500, Proteus Biosciences, 25-6790), at 4 °C. After three washes with 10 mM PBS, samples were incubated at RT for 1 h in secondary fluorescent antibodies (1:1000, diluted in 10 mM PBS). Then, the samples were stained with DAPI (a marker used to stain nuclei). Images were captured with a Leica SP8 confocal fluorescence microscope (Leica Microsystems). The numbers of inner HCs (IHCs) and outer HCs (OHCs) in three turns of the cochleae were counted by ImageJ along the entire cochlear epithelium in each 0.1 mm length of cochlear epithelium. At least three samples for each group were examined.

### Western blot assay

Care was taken to isolate the cochleae under a Zeiss stereomicroscope. Total proteins were prepared using radio immunoprecipitation assay buffer (RIPA, Pierce #89901, solaribo, Waltham MA) with protease and phosphatase inhibitor cocktail (Beyotime, Shanghai, China). Protein concentration was assessed with BCA protein assay (Beyotime, Shanghai, China). Cell (scraping) or tissue samples were homogenized in buffer, followed by centrifugation at 14000 g for 10 min at 4 °C. Supernatants were subjected to western blot analysis by loading same protein in each line. Proteins were fractionated by SDS-PAGE gel electrophoresis and transferred to a PVDF blotting membrane (Millipore, Burlington, MA, USA), subsequently subjected to immunoblotting analysis using appropriate antibodies with proper dilution.

β-Actin (1:1000, HUABIO) and H3 (1:2000, CST) were used as an internal control. Proteins were detected by Super ECL Plus Western Blotting Substrate (BIOGROUND, BG0001), and their intensity was analyzed by ImageJ software (ImageJ software v1.6.0). The primary antibodies used in this study are as follows: anti-LaminB1 (1:1000, CST), anti-H3K4me3 (1:1000, CST), anti-H3K27ac (1:1000, CST), anti-H3K9ac (1:1000, CST), anti-H3K18ac (1:1000, CST), anti-p21 (1:1000, CST) and anti-p16(1:1000, CST). The secondary antibodies include goat anti-mouse antibody (1:5000, ZSGB-BIO) and goat anti-rabbit antibody (1:1000, CST).

### ATAC-Seq and data analysis

ATAC experiment and high-throughput sequencing and partial data analysis were conducted by Seqhealth Technology Co., LTD (Wuhan, China). Two cochlea from one individual was frozen in liquid nitrogen as a biological replicate (three biological replicates in total) and grinded by tissue lyser. The grinded powder was treated with cell lysis buffer, and nucleus was collected by centrifuging for 5 min at 2000 g. Transposition and high-throughput DNA sequencing library were carried out by TruePrep DNA Library Prep Kit V2 for Illumina kit (Catalog NO. TD501, Vazyme). The library products were enriched, quantified, and finally sequenced on NovaSeq 6000 sequencer (Illumina) with PE150 model. Raw sequencing data were first filtered by Trimmomatic (version 0.36) (http://www.usadellab.org/cms/index.php?page=trimmomatic), low-quality reads were discarded, and the reads contaminated with adaptor sequences were trimmed. Clean Reads were further treated with FastUniq (version 1.1) (fastuniq -i dedup_list.xls -o output.or1 -p output.or2) to eliminate duplication. Deduplicated reads were mapped to the reference genome of Mus musculus from ftp://ftp.ensembl.org/pub/release-87/fasta/mus_musculus/dna/ using bowtie2 software (version 2.2.6) (bowtie2 -very-sensitive -X 2000 -x bowtie2_idx -p 10 -1 input.r1 -2 input.r2 -S params.sam) with default parameters. Reads mapped to mitochondria genome were filtered by in-house scripts. The RSeQC (version 2.6) was used for reads distribution analysis [23]. The insert length was counted by Collect Insert Size Metrics tools from picard software (version 2.8.2)(java -jar picard CollectInsertSizeMetrics I = {input} O = {output.insert_length_table} H = {params.insert_length_pic} > {params.Picard_log}). The MACS2 software (Version 2.1.1) (macs2 callpeak -t {input.input_bam} -g {GENOME_SIZE} -n {wildcards.samplename} –outdir {params.out_dir} -f BAM -B –shift -100 –extsize 200 –nomodel –keep-dup all -p 0.01 –SPM) was used for peak calling with the q-value 0.05 cutoff. The bedtools (Version Raw 2.25.0) (intersectBed -a {peak.bed} -b {genome.bed} -wa -wb > {out.bed}) was used for peaks annotation and peak distribution analysis. The quantitative difference in common ACRs was calculated by CSAW, and ACRs with |log2 fold change |≥ 1 and P value < 0.05 were identified differentially enriched ACRs [24]. The Homer (version 4.10) (findMotifsGenome.pl {peak_file} {genome.fa} {out_dir}) was used for motifs analysis [25]. Gene ontology (GO) analysis and Kyoto Encyclopedia of Genes and Genomes (KEGG) enrichment analysis for annotated genes were both implemented by KOBAS software (version: 2.1.1) with a corrected P-value cutoff of 0.05 to judge statistically significant enrichment [26]. Network analysis of TFs (transcription factors) was analyzed by igraph (R package, Version1.0.0) (https://igraph.org/).

### UID mRNA-Seq and data analysis

RNA extraction of cochlea was implemented by TRIzol method. RNA concentration was quantified by NanoDrop, and the integrity of the strip was observed by electrophoresis of 1% agarose gel. Library of UID mRNA-seq quantified by Qubit 2.0 was constructed. If library was qualified by agarose gel electrophoresis, sequencing was performed on Illumina sequencer (Illumina NovaSeq 6000). Raw data and clean data were obtained, and qualification control including the distribution of base mass and analysis of base balance was implemented. Quality control about discarding of duplicating UID data was also conducted. Clean data were mapped to the genome of mice. RPKM (Reads per Kilobase per Million Reads) as an index of gene expression was calculated and normalized. Differentially expressed genes were calculated by edgeR (Version 3.12.1) and filtered by p-value < 0.05 and |log2foldchang|> 1Differential expression analysis of multifactor RNA-Seq experiments with respect to biological variation [27]. KOBAS software was used for KEGG enrichment analysis and Go enrichment analysis for differentially expressed genes.

### Cell culture and treatment

HEI-OC1 cells were cultured in high glucose DMEM culture medium (Gibco, USA) supplemented with 10% fetal bovine serum (FBS) (Gibco, USA) at 33 °C in an incubator containing 10% CO2. Subcultures of cells were performed at 70–80% confluence using 0.25% trypsin EDTA (Gibco, 25,200,056). D-gal (G0750, Sigma) and JQ-1(HY-13030, MedChemExpress) were applied at a concentration of 2–50 mg/mL and 1 μM, respectively, to affect the cells.

### Cell viability assay

The cell viability was detected with the CCK-8 Cell Counting Kit (HY-K0301, MedChemExpress). HEI-OC1 cells were cultured in 96-well plates at 2000 cells/well for 24 h, and then various concentrations of D-gal in DMEM culture medium were added with 6 replicates at least each. At the end of incubation (48 h), 10 μL CCK8 solution was added to each well. Absorbance was measured using an MULTISKAN GO Spectrophotometer (Thermo Scientific) at 450 nm 2 h later. The viability was normalized to the control and all assays were performed at least three times. The half maximal inhibitory concentration (IC50) was calculated using nonlinear regression with a variable slope.

### ROS detection

Cells were seeded in 100 µl media at 3000 cells/well in 96-well black plates with 6 parallel wells in each group and then exposed to D-gal (30 mg/ml) in DMEM culture medium. The plates were further incubated at 33 °C for 48 h. Cells then were loaded with DCFH-DA (S0033S, Beyotime, China), a fluorescent probe for ROS at 10 µM in all wells. After further culture for 30 min in dark, the cells were detected by fluorescence spectrophotometer. All the procedures were done in the dark.

### Quantitative Real-Time PCR (RT-qPCR)

Total RNA were isolated from the cells using TRIzol reagent (TaKaRa Biology Inc., Kusatsu, Shiga, Japan), and cDNA was acquired using MIX reverse transcription primer (TaKaRa Biology Inc.). Real-time quantitative PCR was performed on a LightCycler 480 using SYBR Green (Roche Diagnostics, Basel, Switzerland). The PCRs were performed under the following conditions: predenaturation at 95 °C for 30 s, followed by 40 cycles of denaturation at 95 °C for 15 s, and annealing/extension at 58.8 °C for 20 s. The relative expression of genes was evaluated by using the 2 − ΔΔCt method. Two technical replicates were set for one individual experimental replicate. The primer sequences we used in this study are listed in Table S1.

### Statistical analysis

Data were analyzed using SPSS Statistics 25 software, and GraphPad Prism (version 9.0) was used to perform statistical analyses. One-way ANOVA, two-way ANOVA, or unpaired Student’s t test was performed for comparisons. Values of P < 0.05 were considered significant.

## Results

### Histological characterization of changes in aging cochleae and the development of a mouse model of age-related hearing loss

**Fig. 1:**
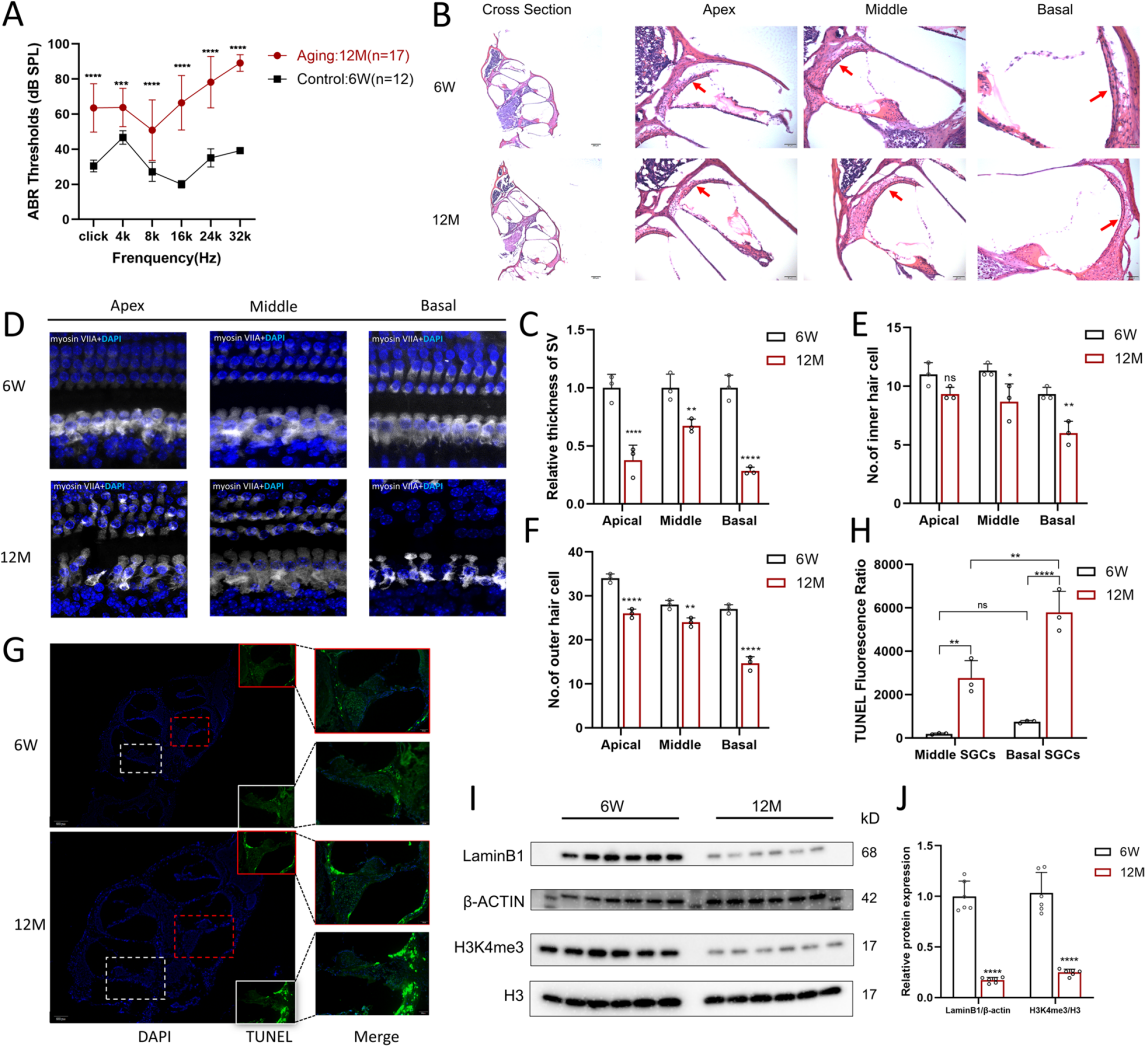
Histological characterization changes in aging cochleae and the identification of an age-related hearing loss mouse model. **A** ABR thresholds were observed in 6 W (n = 12) and 12 M (n = 17) C57BL/6 J mice at 4, 8, 16, 24, 32 kHz and click. **B** Young adult and old aged C57BL/6 J cochlear representative cross sections stained with H&E showed atrophy of SV with age in all three turns. Section thickness is 5 μm. **C** The quantified analysis of SV in three turns, which showed the most SV atrophy appear in basal turns. **D**, **E**, **F** The difference in HCs counts of young adult and old mice at same locations was calculated, and dramatic loss of HCs in cochlear basal turn was observed. (n = 3). **G**, **H** Apoptotic loss of SGCs was detected in two groups and higher TUNEL fluorescence (green color) ratio of SGCs area was detected in 12 M mice than in 6 W mice between the same turn. Blue shows DAPI staining of the nucleus. Scale bar = 50 μm. **I**, **J** Western blot analyses of laminb1 and H3K4me3 in the cochleae from 6 W (n = 6) and12 M (n = 6) mice. The statistical significance was represented as * p < 0.05; **p < 0.01; ***p < 0.001;****p < 0.0001. Bar graph results are means ± SD from 3 independent experiments

In this study, cochleae were collected from six-week-old (6 W) and twelve-month-old (12 M) C57BL/6J mice. The mice evaluated in the experiment received ABR measurements at varying frequencies (4, 8, 16, 24, 32 kHz) and click to determine the level of hearing loss. As shown in Fig. 1A, hearing thresholds were significantly higher (two-way ANOVA, p < 0.0001 or 0.001) in 12 M mice (n = 17) than in 6 W mice (n = 12) at all frequencies and clicks. Interestingly, there were large differences among individual 12 M mice at varying auditory frequencies, consistent with what is observed in humans [5]. Earlier studies have indicated that C57BL/6 J mice display the characteristic pattern of ARHL between the ages of 12 and 15 months, which aligns with our findings mentioned above [28]. We therefore selected the 12 M mice (aging group) and 6 W mice (control group) for subsequent experiments.

Several experiments were performed to identify potential causes of ARHL and confirm that the observed hearing loss was indeed senescence-related. H&E staining of cochlear cross sections revealed age-related atrophy of the SV in all three turns (anatomy shown in Fig. 1B, cross section), as previously noted in prior investigations [29, 30]. Notably, cochlear cross sections showed that among the three turns, the most obvious SV atrophy appeared in basal turns (Fig. 1C), which is consistent with the characteristic high-frequency hearing loss in ARHL. HCs are sensory cells located within the cochlea of the inner ear, the loss of which is a common cause of hearing loss. The dissected cochlear sensory epithelium was stained with anti-myosin 7a antibodies to assess aging-associated HC loss. Obvious loss of IHCs was observed in apical and basal turns (Fig. 1D). A dramatic loss of OHCs in all three turn was observed, especially the basal turn (Fig. 1D). The quantitative analysis showed that HC counts were decreased in the aging group compared with the control group (Fig. 1E, F, n = 3). Progressive apoptotic loss of SGCs in the aged mice was detected by TUNEL staining (Fig. 1G). The TUNEL fluorescence ratio of the SGC area in the same turns showed statistically significant differences between 6 W and 12 M mice (Fig. 1H). TUNEL-positive SGCs were observed in aging mice, whereas most SGCs of control mice were TUNEL-negative. Moreover, a significant increase in the number of apoptotic SGCs in the basal SGC area compared with the middle SGC area was observed in aging mice, differing from the condition in 6 W mice.

Given the lack of specificity of senescence markers, a combination of different markers is always expected [31]. Downregulated levels of LaminB1 (a structural protein of the nuclear lamina) and H3K4me3 (associated with active transcription) have become common markers of senescence [32, 33]. Western blot analysis of cochleae showed that the LaminB1 and H3K4me3 expression levels of the 12 M mice were significantly lower (n = 12, Student’s t test, both p < 0.0001) than those of the 6 W mice (Fig. 1I, J). Based on the above findings, we have fully specified a successful ARHL mouse model.

### Genome-wide chromatin accessibility profiling of the cochleae in ARHL and normal mice

Chromatin accessibility refers to the degree of compactness or openness of the chromatin structure, which can influence the accessibility of regulatory elements, such as enhancers, to transcription factors and other regulatory proteins. To ascertain chromatin-level epigenetic transcriptional regulation, we performed ATAC-seq to construct genome-wide maps of ACRs for the two groups (control group and aging group). Three biological replicates were performed for each group to ensure the consistency and reliability of the results. And the fraction of reads in peaks (FRiP) score was provided for ATAC-seq library quality (Table S2). The similarity within each group is shown by heatmap clustering of Pearson correlation coefficients from the comparison of 6(> 20FRiP)ATAC-seq profiles (Supplementary Fig. S1A). The within-group correlation in the aging and control groups was greater than the between-group correlation, suggesting that the ATAC-seq data are reliable.

**Fig. 2:**
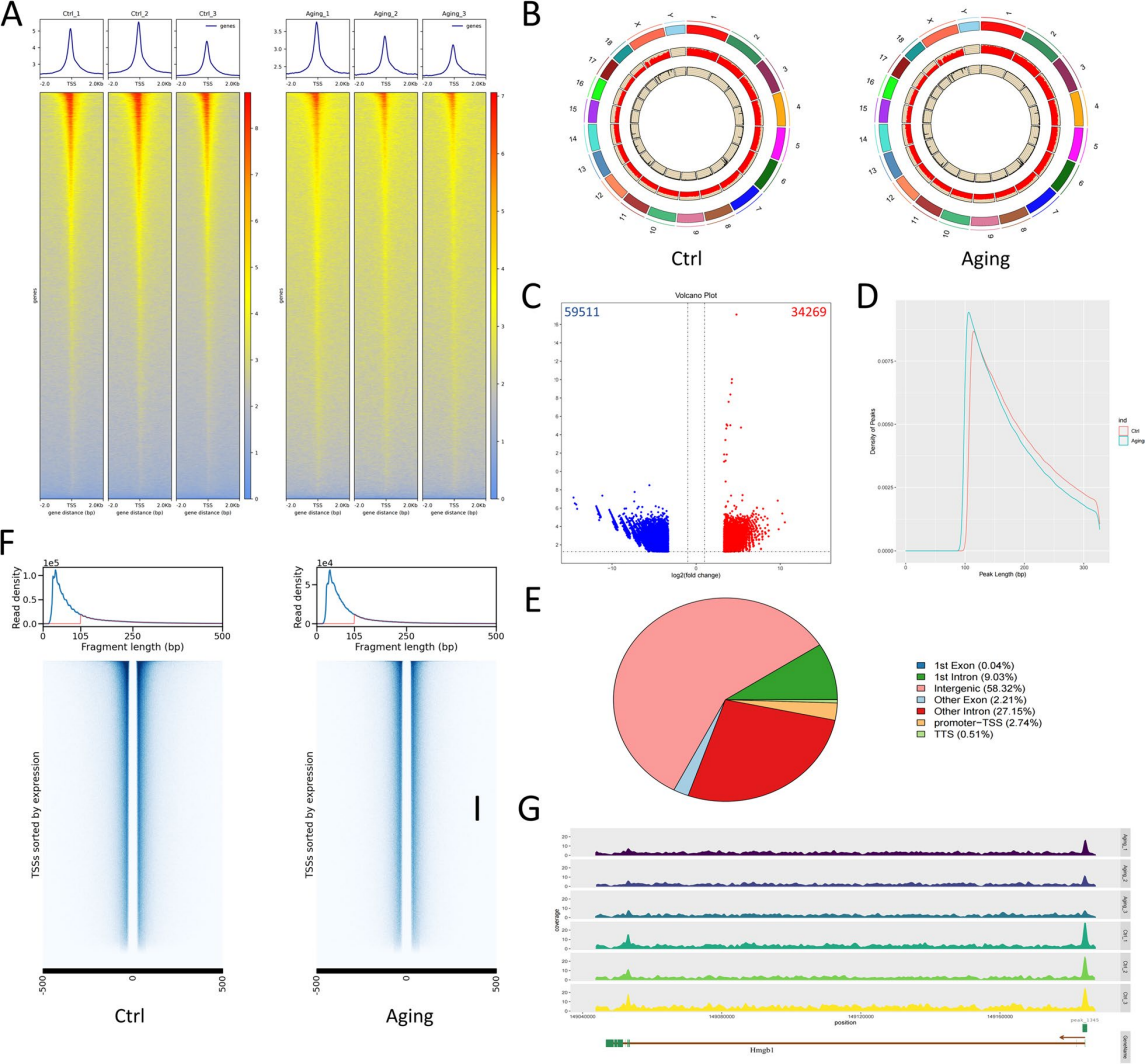
Genome-wide accessible chromatin profiling of cochleae of ARHL and normal mice. **A** Enrichment peaks around the transcription start sites (TSSs), and comparison of the average sequencing depth in TSS of two groups. (X: the distance from the site to the TSS, Y: the average sequencing depth of the site). **B** Genome-wide chromatin accessibility of control and aging cochlea. **C** Volcano plot of differential analysis to the peaks during the aging process of cochlea, which consist of 34,269 increased and 59,511 decreased peaks. **D** The distribution plot of the fragments length revealed that the fragments detected in aging cochleae were shorter overall than in control cochleae. **E** Genomic annotation of identified different ATAC-seq peaks. **F** The enrichment heatmap of nucleosome binding sites revealed a looser or more relaxed chromatin structure in the control cochleae. **G** The geneplot of Hmgb1 in different bioreplicated samples (the arrow indicates the transcription start site and direction, the green region represents the exonic region, and the higher the peak, the stronger the accessibility of that region)

An average of 131.16 million raw reads was obtained from all samples, and more than 98% of these were clean reads (Table S3). The GC content of clean reads in each sample was higher than 40% and showed a normal distribution (Fig. S1B). There were obvious enrichment peaks around the transcription start sites (TSSs), and the average sequencing depth in the TSS of the control group was higher than that of the aging group (Fig. 2A). To detect changes in chromatin accessibility during the cochlear aging process, we performed a differential analysis of the peaks. In total,1,309,220 and 1,249,876 peaks (or chromatin-accessible regions) were identified in control samples and ARHL samples, respectively (Figure S1D), and genome-wide chromatin accessibility of each is shown in Fig. 2B and Figure S1C.93779 peaks were identified as differential peaks (Table S4), which consist of 34,269 increased and 59,511 decreased peaks (Fig. 2C). The distribution plot of peak length revealed that the fragments detected in aging cochleae were shorter overall than those detected in control cochleae (Fig. 2D). These results implied that the overall chromatin accessibility decreased in the aging group during the aging process at the chromosomal level. Genomic annotation of the peaks showed that 2.74% of peaks were located in the promoter regions of genes (± 3 kb of TSSs), 36.18% were in introns, 58.32% were in intergenic regions, and 2.25% were in exons (Fig. 2E). GO and KEGG analyses revealed that the upregulated genes associated with differential peaks between the two groups mainly functioned in the cytoplasm and axons, participating in ‘immune response,’ ‘innate immune deficiency,’‘intestinal immune network IgA production,’ and ‘NF-κB signaling pathway’ (Fig. S2A-B, Table S5-6). GO and KEGG analyses of downregulated genes associated with differential peaks showed that they mainly functioned in the extracellular space and axoplasm, associated with ‘cilium movement,’ ‘innate immune deficiency,’ ‘NF-κB signaling pathway,’ ‘PI3K-Akt signaling pathway,’ and ‘mineral absorption’(Fig. S2C-D, Table S7-8). The x-axis represents the significance of enrichment (represented by -log10pvalue, where a higher value indicates more significant enrichment), while the y-axis represents the enriched terms. Here, we plotted the top 20 most significant terms according to ascending p-values.

Chromatin accessibility is determined by the local nucleosome occupancy and binding of chromatin-related proteins. Active cis-regulatory elements, such as promoters and enhancers, are located in open genomic regions, also known as nucleosome-depleted regions (NDRs). These regions are sensitive to nuclease activity, and thus are also termed DNase I hypersensitive sites, and can be identified by ATAC-seq. In this research, by integrating the structural characteristics of nucleosomes and the distribution of ATAC-seq inserted fragments, fragments within 105 bp are designated as NDRs, and inserted fragments of 105–250 bp are designated as nucleosome distribution areas, allowing precise nucleosome positioning. The heatmap presents the enrichment of nucleosome binding sites on either side of the core binding region of transcription factors in the control and aging cochleae (Fig. 2F). The read density of fragments within 105 bp in the control group was higher than that in the aging group, revealing a looser or more relaxed chromatin structure in the control cochleae, with active gene regulation and transcriptional activity. Additionally, the gene plots of a few well-known senescence markers, including LaminB1, Hmgb1, Egfr, and Mmp2, showed significantly different peaks in their promoter regions (Fig. 2G, Fig. S3A).

### Motif enrichment analysis and motif-TF-gene prediction associated with ARHL progression

Chromatin accessibility plays a key role in determining the binding of transcription factors to regulatory sequences [34]. By analyzing the binding motifs that are enriched within the regulatory regions of genes implicated in ARHL, we can identify potential TFs that may play crucial roles in the disease. Once enriched motifs are identified, motif-TF-gene prediction can be performed to associate TFs with specific ARHL-associated genes.

**Fig. 3:**
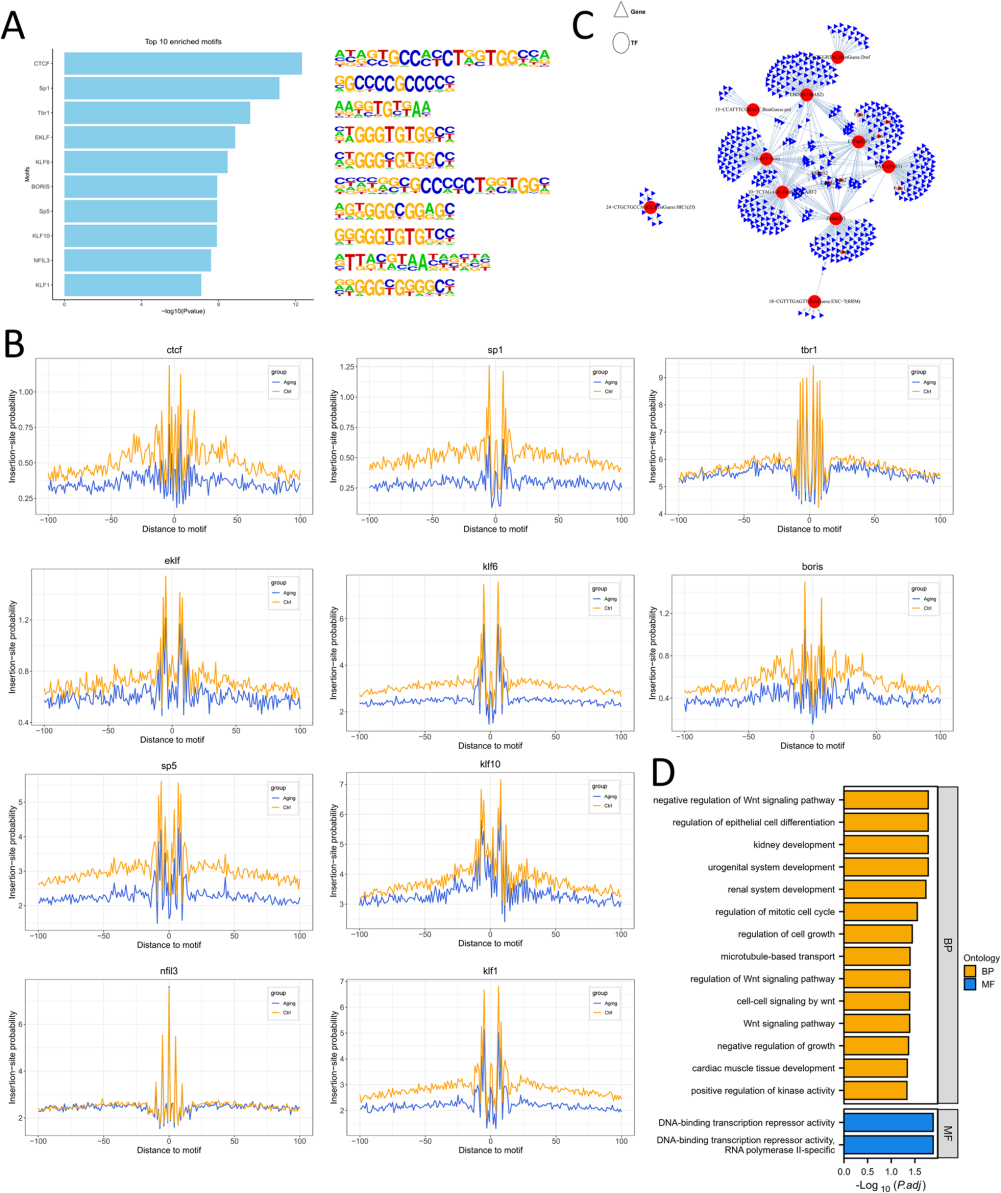
Motif enrichment analysis and motif-TF-gene prediction associated with ARHL progression. **A** The top 10 significantly enriched TF binding motifs by decreased peaks from young to aging according to the enrichment p-values. The corresponding binding motif of TFs is shown. **B** Aggregated footprint plots display the top 10 TFs with reduced footprints, showing an overall footprint for all related transcription factor binding sites for each TF, with individual plots centered on binding motifs. **C** TF-gene regulatory network involved in ARHL progression extracted from HOMER data based on TOP 10 motifs. **D** The top 10 enriched GO terms by the genes regulated by predicted TFs indicated that the predicted TF-regulated genes were significantly enriched in different developmental processes

According to the DNA sequences of different peaks, the top 10 most significantly enriched TF binding motifs were known binding sites for CTCF, SP1, TBR1, ELF, KLF6, BORIS, SP5, KLF10, NFIL3, and KLF1 (Fig. 3A). The significant enrichment of these motifs suggests their importance in regulating gene expression in the context of ARHL. Indeed, during ATAC-seq library construction, DNA sequences that are directly occupied by DNA-binding proteins can be protected and thus less accessible to the transposase enzyme that fragments and tags accessible chromatin regions. This protection leads to the elimination or reduction of ATAC-seq signals within these regions, resulting in a characteristic sequence "footprint" or decreased read density. To further characterize the predicted key TFs involved in ARHL development, we performed a TF footprinting analysis for each TF, which enabled us to identify potential genome-wide TF binding events. The results of the analysis demonstrated a noticeable change in footprints across the 10 TFs studied (Fig. 3B). Specifically, the footprints of all 10 TFs displayed a deeper, more pronounced pattern in the control group than in the aging group, revealing that there were alterations in the protein‒DNA interactions and accessibility of the chromatin regions between the two groups. The increased footprint size or insertion-site probability indicates a higher likelihood of transposase enzyme insertion events occurring in the control group compared to the aging group. This suggests that there is a higher degree of protection for DNA sequences bound by TFs in the control group, resulting in increased accessibility and increased transposition during ATAC-seq library construction.

In order to study possible TF binding events in different periods, motif analysis was carried out on identified difference accessibility peaks, and matching binding TFs were extracted from HOMER data based on TOP 10 motifs, thus obtaining the regulatory network diagram of TFs and genes associated with difference peaks involved in ARHL progression (Fig. 3C, Table S9). Motif-TF-gene prediction analysis can help uncover potential TFs that may interact with these motifs and regulate the expression of ARHL-related genes. Each TF is represented as a red node in the network, and the edges between the nodes represent potential regulatory relationships between the TFs and target genes. The GO-KEGG pathway enrichment analysis of the TF network indicated that the predicted TF-regulated genes were significantly enriched in different developmental processes (such as urogenital system, renal system, and cardiac muscle tissue development and regulation of cell growth), regulation of kinase activity, DNA-binding transcription repressor activity, and negative regulation of the Wnt signaling pathway (Fig. 3D). This suggests that the TFs in the network may be involved in controlling key signaling pathways and transcriptional regulatory mechanisms through the regulation of kinase activity and repressing gene transcription.

### Genome-wide gene transcriptional analysis comparing control and aging cochleae and prediction of ARHL-related TFs

**Fig. 4:**
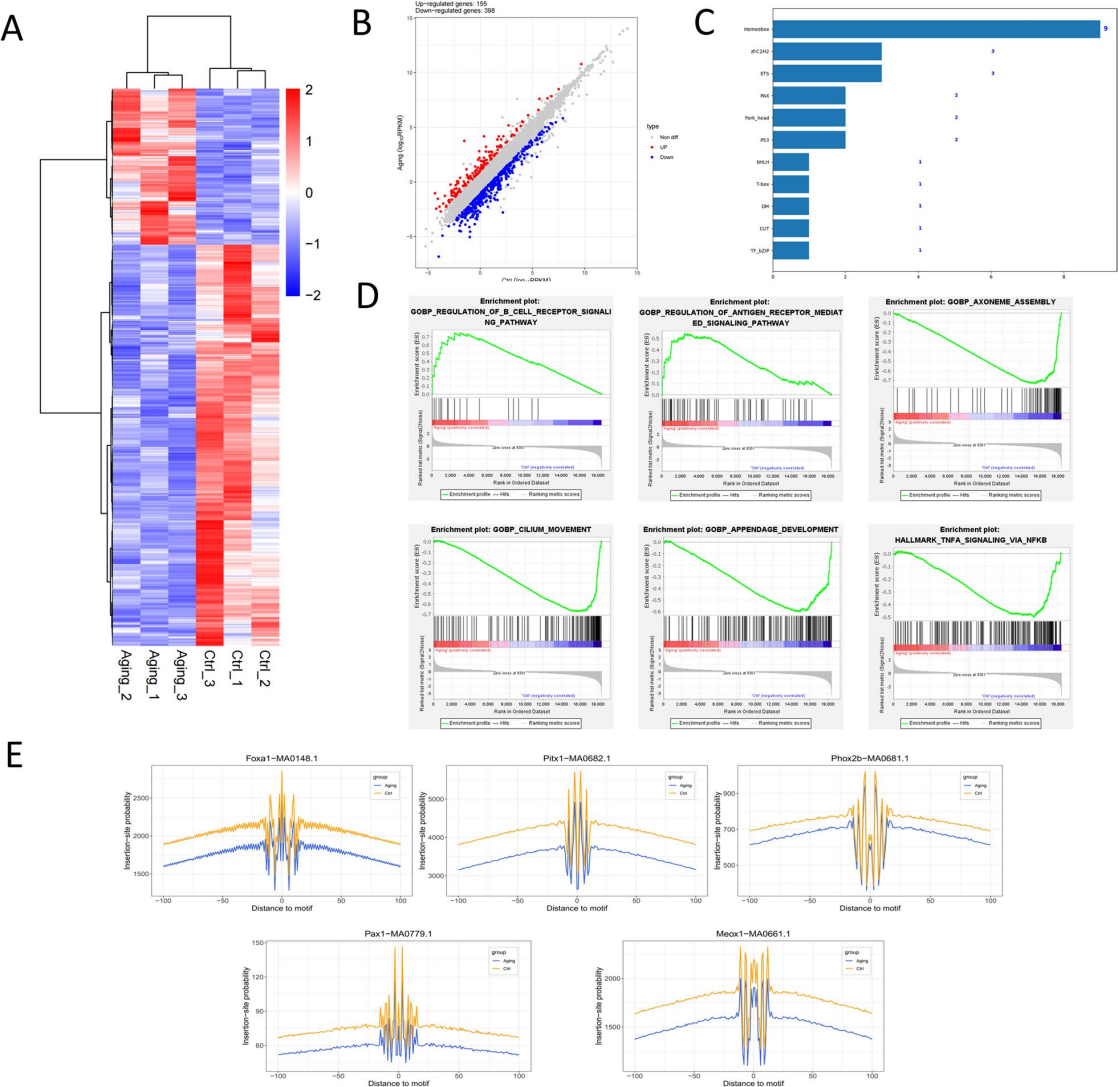
Genome-wide gene transcriptional difference analysis between the young and aging cochleae and TFs prediction related to ARHL. **A** Heatmap analysis and hierarchical clustering on transcriptome samples using normalized read counts (TPM values). Each row represents a gene, and the TPM values were Z-scaled by row. The scale bar indicates the Z-scaled TPM values. **B** The scatter plot of differential expression genes. Gray dots represent genes that are not differentially expressed, blue dots represent genes that are differentially downregulated, and red dots represent genes that are differentially upregulated. **C** GSEA enrichment analysis of transcriptome with high enrichment scores. **D** Differential binding TFs prediction using the transcriptomic data. **E** The footprint of top 5 TFs according to the |logFC| was all significantly decreased from young to aged mice genome-wide

To identify global gene expression changes between the two groups, we performed RNA sequencing using the cochlear tissues of three pairs of control and aging mice. An average of 35.75 million clean reads (accounting for 84.04%, Table S10) and a 98.54% mapping ratio were obtained from our deep RNA-seq libraries (Table S11). Hierarchical clustering based on gene expression levels divided the samples into two distinct clusters (red: upregulated, blue: downregulated) corresponding to the control and aging mice (Fig. 4A). In addition, we provide a higher-resolution display of the genes with significant differences between the two clusters (including gene names) in the original image for a more intuitive, in-depth analysis of the gene expression patterns and key references (Fig. S4). Differential analysis identified 155 and 399 significantly upregulated and downregulated genes, respectively (Fig. 4B, Table S12). These data reveal transcriptome changes during the development of ARHL. GSEA of 18,381 genes showed that 8301 (45.2%) genes with a correlation area of 38.5% were more highly expressed in aging cochleae, and 10,080 (54.8%) genes with a correlation area of 61.5% were more highly expressed in control cochleae. Then, we found that 1731 gene sets were upregulated in the aging group and 3442 gene sets were upregulated in the control group. GSEA enrichment results identified regulation of the B-cell receptor signaling pathway, regulation of the antigen receptor-mediated signaling pathway, axoneme assembly, appendage development, and cilium movement as gene sets with high enrichment scores (Fig. 4C). Among these gene sets, the first two were significantly enriched in the aging group, while the last three were significantly enriched in the control group. In addition, we found that the gene sets of various developmental processes, WNT–β-catenin signaling, ciliary plasm, and TNFA signaling via NFKB were also significantly enriched, which corresponded to the GO enrichment results of ATAC-seq. To explore the potential TFs related to the cochlear aging process, we performed TF prediction using the transcriptomic data (Table S13). Among all the predicted TFs, 9 were from the homeobox family, 3 were from the Fz-C2H2 family, and 3 were from the ETS family (Fig. 4D). The analysis of footprint changes of the top 5 TFs (|log fold change|> 1.8), including Phox2b, Pitx1, Pax1, Foxa1, and Meox1, indicated that the footprints of the 5 TFs were significantly decreased from young to aged mice genome-wide, which was in accord with the TF predictions from the transcriptomic data (Fig. 4E).

### Integrated analysis of chromatin accessibility and gene expression during ARHL progression

**Fig. 5:**
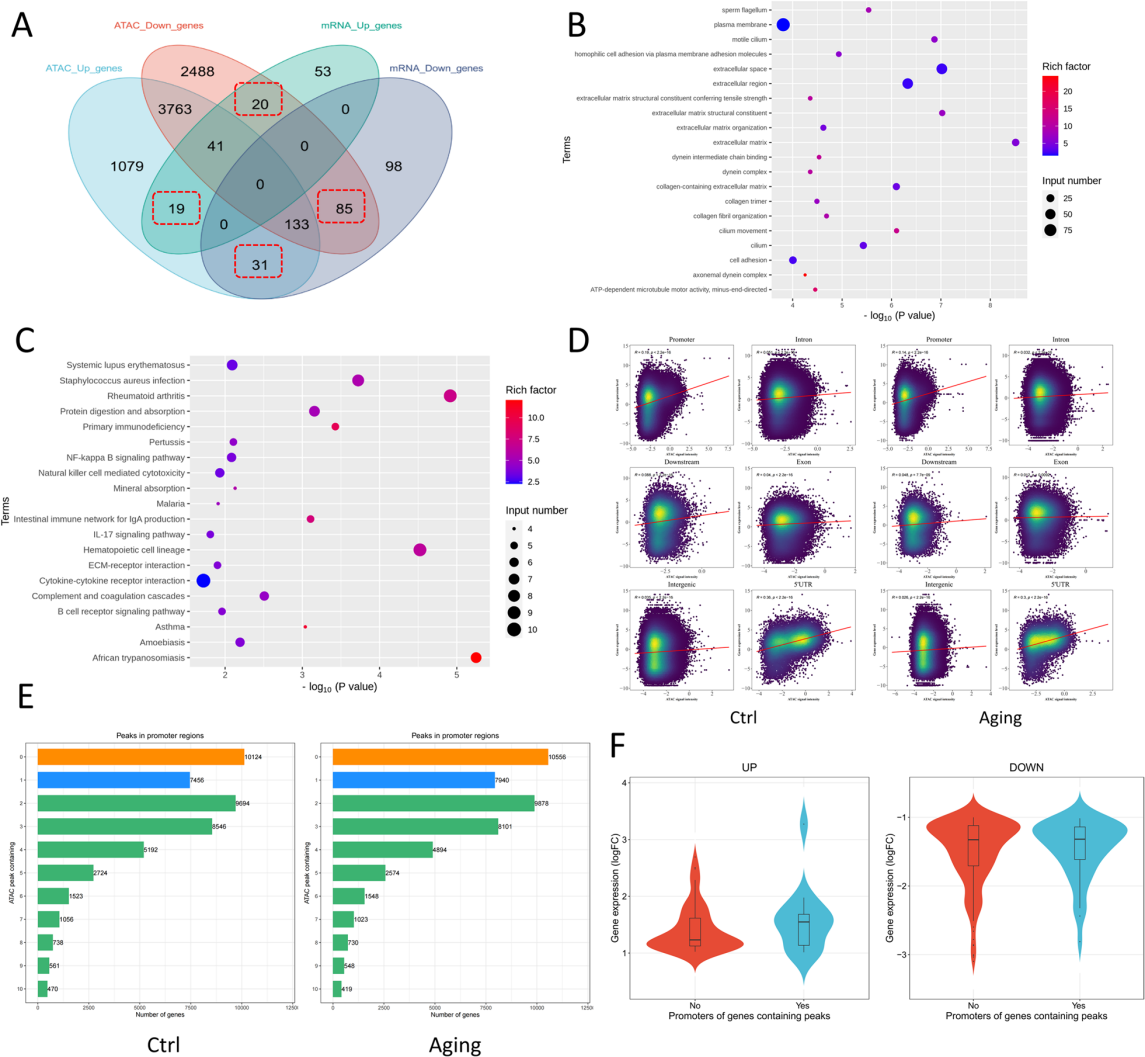
Integrated analysis of chromatin accessibility and gene expression during ARHL development. **A** Venn diagram showing the number of genes in different ATAC and mRNA state, providing a more reliable analysis of the regulatory mechanisms underlying age-related changes in gene expression. **B** GO enrichment analysis of differentially expressed genes with different peaks. **C** KEGG analysis of differentially expressed genes with different peaks. **D** Gene element-based correlation analyses between peak intensity and gene expression, suggesting the importance of chromatin accessibility in the promoter and 5’UTR regions for proximal regulation. R is Pearson’s correlation coefficient and P is the significance test for the correlation coefficient. **E** The number of genes containing different numbers of peaks in the promoter regions in two groups. Most genes in both groups have 1–3 peaks in their promoter regions. **F** Comparing expression distribution of up or downregulated genes based on whether they contain promoter peaks or not

To address the potential discrepancy between chromatin accessibility and gene expression levels, a Venn chart analysis was employed to ensure that changes in gene expression were specifically associated with alterations in chromatin accessibility at different gene regions. By focusing on the overlapping regions, we are able to more confidently attribute gene expression changes to specific alterations in the chromatin landscape, providing a more reliable analysis of the regulatory mechanisms underlying age-related changes in gene expression. Nineteen all-up genes and 85 all-down genes were considered directly linked to variations in chromatin accessibility (Fig. 5A, Table S14). In contrast, 20 ATAC-down with mRNA-up genes and 31 ATAC-up with mRNA-down genes would be considered influenced by other factors, such as RNA editing, RNA splicing, and posttranscriptional modifications, rather than changes in chromatin openness. The GO enrichment analysis showed that the assembly and movement of the cilium were necessary for hearing maintenance, reflecting the important role of HCs in ARHL progression (Fig. 5B). The KEGG pathway analysis emphasized the potential role of NF-κB pathway signaling (Fig. 5C).

To explore the regulatory relationship between chromatin accessibility and gene transcription, we initially conducted an analysis to examine the correlation between the intensity of chromatin accessibility peaks and the expression levels of nearby genes in two distinct groups of mice.

Correlation analysis (Pearson’s correlation coefficient) revealed that peaks in different positions of genes exhibited distinct effects on the regulation of nearby genes. In the control group, the peaks in the intron, exons, and intergenic regions showed the weakest effect on gene expression (intron: R = 0.051; exon: R = 0.04; intergenic regions: R = 0.035; downstream: R = 0.26). The peaks in the 5’UTRs and promoter regions showed a moderate effect on gene expression (5’UTRs: R = 0.36; promoters: R = 0.19, p value < 0.0001) (Fig. 5D). Similar to the case in the aging group, the peaks in the intron, exons, and intergenic regions showed the weakest effect on gene expression (intron: R = 0.032; exon: R = 0.012; intergenic regions: R = 0.026; downstream: R = 0.048), while the peaks in the 5’UTRs and promoter regions showed a moderate effect on gene expression (5’UTRs: R = 0.3; promoters: R = 0.14, p value < 0.0001) (Fig. 5D). The correlation analyses between chromatin accessibility and gene expression suggested the importance of chromatin accessibility in the promoter and 5’UTR regions for proximal regulation, providing insights into the differential regulatory mechanisms associated with distinct chromatin accessibility patterns. We further comprehensively explored the changes in open chromatin in the promoter regions of aging and control cochleae. In control cochleae, 10,124 genes had no peak (n peak = 0), 7456 genes contained a single peak (n peak = 1), and 30,504 genes contained multiple peaks (n peak > 1) in their promoter regions (Fig. 5E). Most genes in both groups have 1–3 peaks in their promoter regions. In aging cochleae, 10,556 genes had no peak (n peak = 0), and 7940 genes contained a single peak (n peak = 1), but 29,715 genes contained multiple peaks (n peak > 1) in their promoter regions. Most upregulated genes containing 0 peaks showed lower expression, while most genes containing peak(s) showed higher expression; most downregulated genes did not contain peaks and showed more obvious fold changes in expression than those with peak(s) (Fig. 5F).

### Identification of SASEs and prediction of their target genes in mice with ARHL

**Fig. 6:**
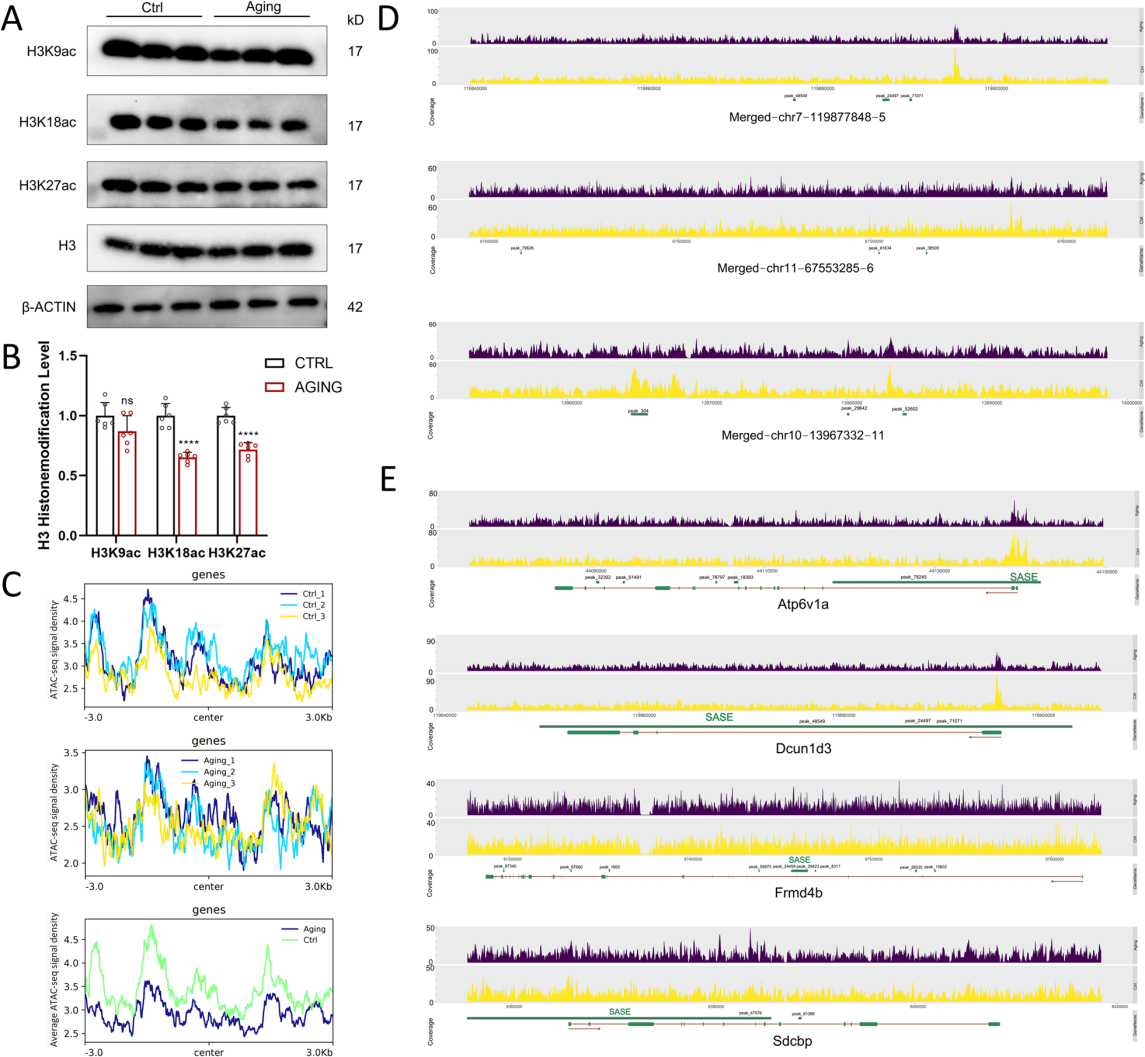
Identification of SASEs and prediction of their target genes in mice with ARHL. **A**, **B** Western blot analyses of H3K9ac, H3K18ac, and H3K27ac in the cochleae of 6 W and12 M mice. The statistical significance was represented as * p < 0.05; **p < 0.01; ***p < 0.001;****p < 0.0001. Bar graph results are means ± SD from 6 independent experiments. **C** The distribution of SASEs region peaks around the transcription start sites and average ATAC-seq signal density in the young state and aging state of cochlea surrounding the SASE regions. **D** Geneplots of identified SASEs. The position of different peaks was labeled with green cubes and peak numbers. **E** Geneplots of SASE-linked genes. The position of different peaks and gene exons was marked with green cubes. The thick green line represents the location of SASE

Histone acetylation plays a crucial role in modulating chromatin accessibility. Acetylated histones can create a more open chromatin structure, facilitating the recruitment of transcription factors and other regulatory proteins to specific genomic regions. To further explore the potential reasons for chromatin accessibility changes in the aging cochleae, we screened the global status of the most common histone acetylation modifications, including H3K9ac, H3K18ac, and H3K27ac. Western blot analysis showed that the levels of H3K27 and H3K18 acetylated histones were decreased in aging cochleae compared to control cochleae (n = 6, Student’s t test, both p < 0.0001), while no significant alteration was detected in H3K9ac (Fig. 6A, B). H3K27ac is a specific histone modification that plays a crucial role in gene regulation and is often found in regions of the genome where gene transcription is highly active. SEs, on the other hand, are large clusters of transcriptional enhancers that drive expression of genes defining cell identity and often physically interact with the promoter region of their target genes, forming chromatin loops and enhancing gene transcription. They are characterized by high levels of H3K27ac and other chromatin marks associated with active transcription, such as mediator complex binding and the presence of transcription factor binding sites [35]. The presence of H3K27ac at SEs reflects their active and highly accessible chromatin state, allowing robust and sustained gene expression. CTCF can bind to specific DNA motifs and create topology associating domains (TADs), which are chromatin loops that restrict the contacts that a promoter makes with distal elements such as enhancers within the loop [36]. The motif analysis indicated that CTCF is the TF most frequently bound to its motif in these samples (i.e., the top 1), also indicating that SEs may play a crucial role in ARHL. Finally, we found that SASEs (senescence-associated SEs) tended to be involved in the process of ARHL development.

A H3K27ac chromatin immunoprecipitation and sequencing study conducted by Sturmlechner and his colleagues identified 40 common SASEs and 50 transcriptionally activated genes that are associated with these common SASEs in senescent MEFs with senescence induced through three distinct stressors [16]. Other studies have shown that SEs are generally enriched in regions of accessible chromatin; moreover, ATAC-seq has been widely conducted to monitor chromatin accessibility in SE regions and has confirmed that SEs are indeed associated with the phenotype studied in the active or inactive state [37]. First, we drew the peak center plots for all SE region peaks, referring to the known SASE list. As expected, the SE region peaks showed a consistent distribution among the biological replicates, which revealed the high conservation of SASEs related to ARHL (Fig. 6C). The merged plot revealed that the sequencing depth of different SE regions was overall higher in the control group than in the aging group, which indicated that the SASE regions of control cochleae were in a more active and highly accessible chromatin state, allowing the TFs and regulatory factors to bind. In total, we identified 18 SASEs with different peak numbers (n ≥ 1) from the ATAC data and 18 candidate SASE genes. The list of the identified SASE IDs and candidate SASE genes is provided in Table 1. Figure 6D and Figure S5 show the plots of genes associated with candidate SASEs and display the gene plots of SASEs (different peak numbers ≥ 2), which could provide valuable insights into the molecular mechanisms underlying the development and progression of ARHL in mice. The potential functional SE regions of candidate SASE genes were observed in their gene plots, labeled as green squares with peak numbers (Fig. 6E). The identification of SASEs and the prediction of their target genes in ARHL mice could contribute to our understanding of the regulatory networks involved in age-related hearing loss.

**Table 1:** Identified SASEs related to ARHL with different peaks number and candidate SASE genes

Peaks number	ARHL-related SASE ID	Chr	Start	End	Annotation	SASE genes within +/−50 kb	Candidate SASE genes
3	Merged-chr7-1198778485	chr7	119850046	119903375	Intron (NM_001163703, intron 1 of 2)	2610020H08Rik, Dcun1d3, Lyrm1	Abca1
3	Merged-chr11-67553285-6	chr11	67455676	67601402	Intergenic	Gas7	Atp6v1a
3	Merged-chr10-13967332-11	chr10	13963003	13988572	Intron (NM_010437, intron 1 of 8)	Hivep2	Best1
2	Merged-chr6-97823414-3	chr6	97815138	97831338	Intron (NM_001113198, intron 1 of 9)	Mitf	Dcun1d3
2	Merged-chr6-97460802-1	chr6	97456558	97465046	Intron (NM_145148, intron 2 of 23)	Frmd4b	Eva1c
2	Merged-chr4-6369737-5	chr4	6350781	6385699	Intron (NM_016807, intron 1 of 8)	Nsmaf, Sdcbp	Fam102b
2	Merged-chr17-39845387-2	chr17	39842779	39849041	Noncoding (NR_046233, exon 1 of 1)	Rn45s	Frmd4b
1	Merged-chr1-133026055-3	chr1	133017865	133034168	Promoter-TSS (NR_126506)	Mdm4, Pik3c2b	Fth1
1	Merged-chr14-73385423-3	chr14	73369749	73396062	Intron (NM_008410, intron 1 of 5)	Itm2b, Rb1	Gas7
1	Merged-chr16-27394277-2	chr16	27386586	27401986	intron (NM_001289436, intron 1 of 11)	Ccdc50, Ostn, Uts2b	Hivep2
1	Merged-chr16-44131633-6	chr16	44118035	44142344	Intron (NM_007508, intron 1 of 14)	Atp6v1a, Naa50, Usf3	Itm2b
1	Merged-chr16-90934761-1	chr16	90932413	90937110	Promoter-TSS (NM_026502)	1110004E09Rik, Eva1c, Synj1	Lrrc57
1	Merged-chr19-9946091-1	chr19	9936121	9956061	Intergenic	Best1, Fth1, Incenp, Rab3il1	Lyrm1
1	Merged-chr2-120641133-1	chr2	120628850	120653417	Intergenic	Haus2, Lrrc57, Snap23	Mitf
1	Merged-chr2-73040067-3	chr2	73023408	73050972	Intergenic	Ola1, Sp3, Sp3os	Rab3il1
1	Merged-chr3-109019468-2	chr3	109007777	109033660	Intron (NM_001163567, intron 1 of 10)	Fam102b, Henmt1, Slc25a54	Rb1
1	Merged-chr4-53131601-1	chr4	53102977	53160225	Intron (NM_013454, intron 3 of 49)	Abca1	Sdcbp
1	Merged-chr9-113707374-1	chr9	113705891	113708857	Intron (NM_001164678, intron 1 of 17)	Clasp2, Pdcd6ip	Sp3os

### Changes in the expression levels of SASE genes in D-gal-induced aging and JQ-1-treated HEI-OC1 cells

**Fig. 7:**
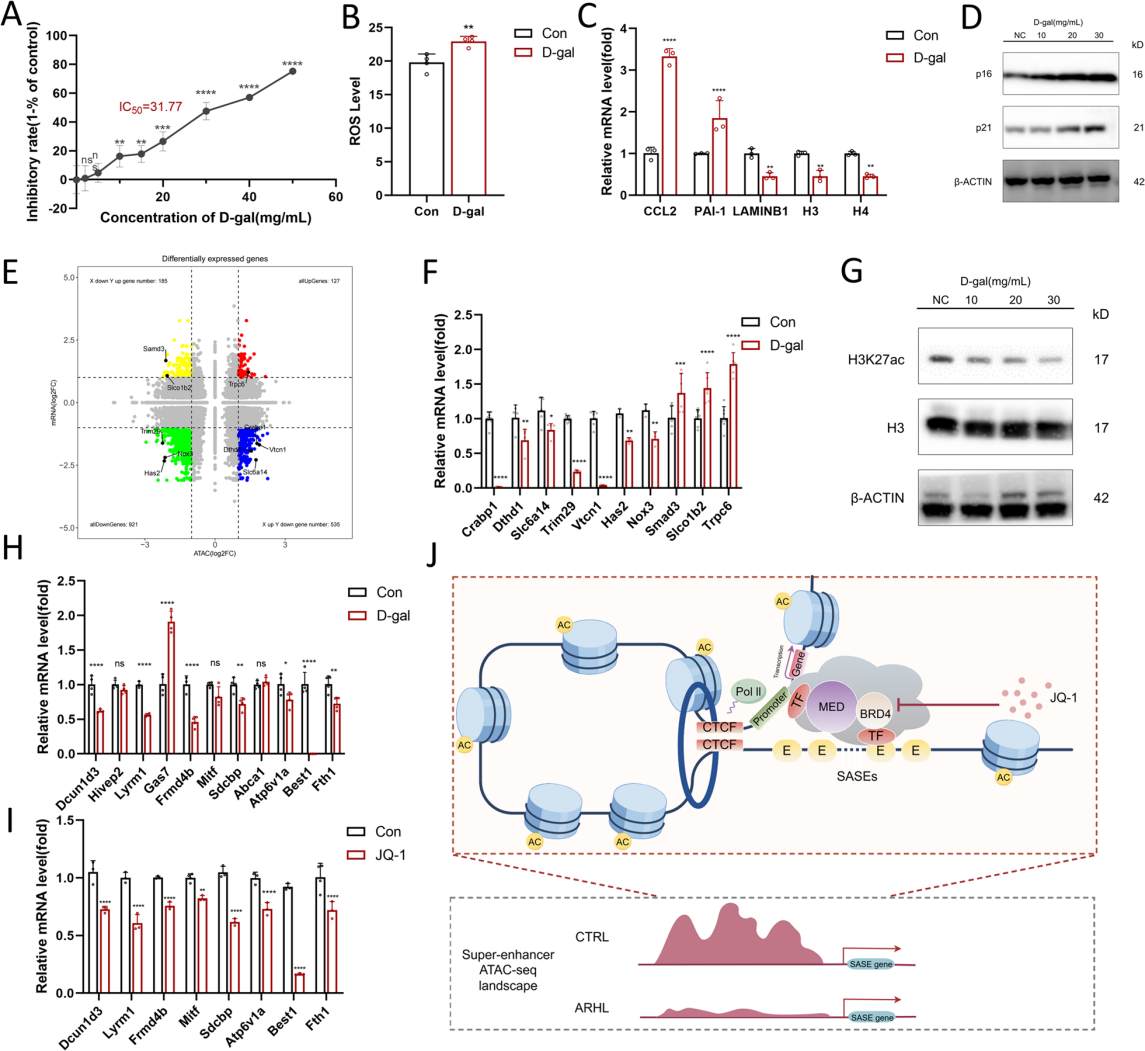
The expression level changes of SASE genes in D-gal-induced aging and JQ-1-treated HEI-OC1 cells. **A** The CCK8 results showed that the inhibitory rate of HEI-OC1 cells rises as the increased D-gal concentration (n = 6). **B** The ROS level increased after the HEI-OC1 treated by D-gal (n = 4). **C** The mRNA level of aging hallmarks in the control and D-gal treated cells (n = 3). **D** The protein level of p16, p21 increased as the D-gal concentration increased. **E** The differential expressed genes with different chromatin accessibility state. **F** The mRNA level changes of the top genes in different four quadrants between normal and mimic-aging cells (n ≥ 4). **F** The levels of H3K27ac decreased with increasing D-gal concentration. **H** The mRNA level changes of the SASE-linked genes in D-gal-induced aging cells (n ≥ 3). **I** The mRNA level changes of the SASE-linked genes after treated by JQ-1(1 μm) (n ≥ 3). **J** Schematic diagram of possible mechanisms underlying positive role of identified SASEs in ARHL (drawn by Figdraw). During the aging process of the cochlea, the positive regulation of candidate SASE gene expression is inhibited due to decreased chromatin accessibility, and inhibition of SE activity caused by JQ-1 could lead to similar effects. The statistical significance was represented as * p < 0.05; **p < 0.01; ***p < 0.001;****p < 0.0001

The most common type of ARHL, sensory presbycusis, involves damage to the HCs located at the base of the cochlea. Treating cells or animals with D-gal is a common method used to induce cellular and animal aging for studies of the effects of aging on various biological processes [38]. To investigate the role of candidate SASE genes in the aging of mouse cochlear HCs, we established an aged mouse cochlear HC model by treating HEI-OC1 cells (derived from conditionally immortalized mouse auditory cells) with different D-gal concentrations (2, 5, 10, 15, 20, 30, 40, and 50 mg/mL) for 48 h. CCK8 assay results showed that the cell density began to decrease when the concentration of D-gal was higher than 2 mg/ml, and the cell density was significantly decreased when the D-gal concentration was 10 mg/ml or higher (Fig. 7A, p < 0.01, n = 6). The IC50 of D-gal was 31.77 mg/ml, and 30 mg/ml was considered a suitable concentration to induce cellular aging. DCFH-DA was used to detect the ROS levels in cells with D-gal-induced aging, and fluorescence intensity results showed that the ROS level was significantly increased compared to controls when the D-gal concentration was 30 mg/mL (Fig. 7B). Other hallmarks of aging are decreases in H3, H4, and LaminB1 [39], a series of protein known to contribute to cellular senescence, as indicated by increased C–C motif chemokine ligand 2 (Ccl2) [40], C–C motif chemokine ligand 20(Ccl20) [41], and plasminogen activator inhibitor-1(PAI-1) [42] expression (Fig. 7C). The expression levels of p16 and p21 (both common senescence indicators) increased with increasing D-gal concentration in aging HEI-OC1 cells (Fig. 7D), which indicated that the degree of aging had a positive relationship with increasing D-gal concentration. The four-quadrant analysis for ATAC-seq and RNA-seq showed 185 ATAC-down and mRNA-up genes, 535 ATAC-up and mRNA-down genes, 127 all-up genes and 921 all-down genes (Fig. 7E). We examined the expression-level changes in the top genes in different quadrants between normal and model cells (Fig. 7F) and labeled them in Fig. 7E; the findings were in general accord with our expectations.

Interestingly, we also found that the levels of H3K27ac decreased with increasing D-gal concentration, which revealed that there was overall less chromatin accessibility and less active enhancers in aging cells (Fig. 7G). To investigate the role of SASEs in the aging process of these cells, we evaluated the expression levels of 11 candidate SASE genes. We found that all of them were changed, and 8 of them significantly decreased (Fig. 7H). Based on the above, we hypothesized that the positive regulation of candidate SASE gene expression was inhibited due to decreased chromatin accessibility during the aging process of the cochlea and that the suppression of SE activity could elicit similar responses. SEs are characterized by their larger size, higher transcription factor occupancy, and increased binding of coactivators such as BRD4 and the mediator complex. BRD4, a member of the bromodomain and extraterminal domain (BET) family of proteins, plays a significant role in transcription regulation and chromatin remodeling. JQ-1 is a specific and potent BRD4 inhibitor that can interfere with the recruitment of transcriptional coactivators and other factors necessary for gene expression controlled by active enhancers and SEs [43]. To confirm the regulatory relationship of SASEs with their related genes, HEI-OC1 cells were treated with 1 μM JQ-1 for 48 h. As shown in Fig. 7I, blocking BRD4 led to a slightly greater reduction in the expression level of SE-linked genes compared to that in control cells, including Atp6v1a, Scdcbp, Dcun1d3, Mitf, among others. All eight candidate SASEs and their nearby peaks with different chromatin accessibility are shown in Table 2. Taken together, these data from an aged mouse cochlear HC model reinforce the important roles of candidate SASEs and their related genes in the aging process of HEI-OC1 cells.

**Table 2:** The chromatin accessibility changes of candidate genes nearby identified SASEs involved in the aging of HEI-OC1 cells (the bold text represents the occurrence within the identified SASE segment)

GeneID	Seqnames	Start	End	Width	Peakid	Strand	logFC	PValue	FDR	Annotation
Dcun1d3	7	**119887521**	**1.2E + 08**	**712**	**peak_24497**	**–**	**− 1.457358865**	**0.004943589**	**0.20483498**	**intron (ENSMUST00000059851, intron 1 of 2)**
		**119877221**	**1.2E + 08**	**228**	**peak_48549**	**–**	**− 1.073696622**	**0.013266131**	**0.24954676**	**intron (ENSMUST00000059851, intron 1 of 2)**
		**119890625**	**1.2E + 08**	**196**	**peak_71071**	**–**	**1.036684158**	**0.024994521**	**0.28281603**	**intron (ENSMUST00000059851, intron 1 of 2)**
Lyrm1	7	**119919109**	**1.2E + 08**	**337**	**peak_27750**	** + **	**− 1.052558082**	**0.005950005**	**0.21317979**	**intron (ENSMUST00000207270, intron 4 of 4)**
		119935578	1.2E + 08	144	peak_45801	+	**− **1.322620431	0.011907855	0.24088924	intron (ENSMUST00000207270, intron 4 of 4)
		**119933318**	**1.2E + 08**	**111**	**peak_85078**	** + **	**− 1.07753413**	**0.038715747**	**0.31505057**	**intron (ENSMUST00000207270, intron 4 of 4)**
		**119907848**	**1.2E + 08**	**113**	**peak_87543**	** + **	**1.13836622**	**0.04252096**	**0.32906334**	**intron (ENSMUST00000207270, intron 1 of 4)**
Sdcbp	4	**6383145**	**6384137**	**993**	**peak_47576**	** + **	**− 1.644684407**	**0.012844813**	**0.24763918**	**intron (ENSMUST00000029912, intron 4 of 8)**
		**6388471**	**6388703**	**233**	**peak_61366**	** + **	**1.203629035**	**0.019397438**	**0.27249778**	**intron (ENSMUST00000029912, intron 6 of 8)**
Mitf	7	**97819004**	**97819322**	**319**	**peak_10226**	** + **	**− 1.156204588**	**0.001603607**	**0.16659097**	**intron (ENSMUST00000203884, intron 1 of 9)**
		**97824775**	**97825072**	**298**	**peak_42298**	** + **	**1.027959371**	**0.010629002**	**0.2352373**	**intron (ENSMUST00000203884, intron 1 of 9)**
		97811164	97811277	114	peak_60837	+	1.403320827	0.019084679	0.27085343	intron (ENSMUST00000203884, intron 1 of 9)
		97831458	97832153	696	peak_78462	+	1.073133859	0.030989079	0.3013727	intron (ENSMUST00000203884, intron 1 of 9)
Frmd4b	9	97355250	97355411	162	peak_1605	–	**− **1.724341886	0.00017192	0.11271064	intron (ENSMUST00000113355, intron 6 of 23)
		97469433	97469543	111	peak_8317	–	**− **1.912430345	0.001244113	0.15814249	intron (ENSMUST00000113355, intron 2 of 23)
		97535464	97535698	235	peak_15602	–	**− **1.389768399	0.002706149	0.18083641	intron (ENSMUST00000113355, intron 1 of 23)
		**97456241**	**97456718**	**478**	**peak_24459**	**–**	**− 1.600663964**	**0.004931453**	**0.20468066**	**intron (ENSMUST00000113355, intron 2 of 23)**
		97524812	97525299	488	peak_26535	–	1.317813254	0.00555457	0.20984648	intron (ENSMUST00000113355, intron 2 of 23)
		**97464587**	**97464758**	**172**	**peak_29423**	**–**	**1.248364148**	**0.006487548**	**0.21758593**	**intron (ENSMUST00000113355, intron 2 of 23)**
		97438169	97438293	125	peak_59975	–	**− **1.193937512	0.018611958	0.26833359	intron (ENSMUST00000113355, intron 2 of 23)
		97296802	97297023	222	peak_67340	–	1.015504514	0.023241265	0.27861708	intron (ENSMUST00000113355, intron 21 of 23)
		97334152	97334275	124	peak_67660	–	1.014563179	0.023419903	0.27884967	intron (ENSMUST00000113355, intron 8 of 23)
Atp6v1a	16	44106461	44106881	421	peak_18393	–	1.106955189	0.003422783	0.19259704	intron (ENSMUST00000114666, intron 8 of 14)
		44090307	44090574	268	peak_32302	–	1.098457768	0.007192549	0.21758593	intron (ENSMUST00000114666, intron 13 of 14)
		44093427	44093583	157	peak_61491	–	1.20228798	0.019487901	0.27305913	intron (ENSMUST00000114666, intron 12 of 14)
		**44127794**	**44127954**	**161**	**peak_78245**	**–**	**− 1.119216679**	**0.030831688**	**0.30111969**	**intron (ENSMUST00000114666, intron 1 of 14)**
		44104279	44104397	119	peak_78797	–	**− **1.114554648	0.031266468	0.30193883	intron (ENSMUST00000114666, intron 8 of 14)
Best1	19	**9984911**	**9985020**	**110**	**peak_24845**	** + **	**− 1.317559258**	**0.005073366**	**0.20660848**	**exon (ENSMUST00000025563, exon 4 of 4)**
Fth1		**9984911**	**9985020**	**110**	**peak_24845**	** + **	**− 1.317559258**	**0.005073366**	**0.20660848**	**exon (ENSMUST00000025563, exon 4 of 4)**

## Discussion

More recently, the primary role of mutations as a driver of aging has been questioned. In the 1950s, Szilard and Medawar separately suggested that aging is the result of genetic information loss stemming from DNA damage [44]. Several studies have found that many types of aging cells have a low number of mutations [45, 46], and mammals can be cloned from aged somatic cells to create new individuals with normal lifespans [47]. These findings indicate that aging involves an intricate interplay between the cellular machinery and the information stored in the genome and epigenome, often referred to as biological ‘hardware’ and ‘software’ [48]. Similarly, age-related diseases, including ARHL, are the result of the complex interplay of many factors, including both intrinsic and extrinsic factors. Among the extrinsic factors, epigenetic inheritance plays a significant role. In this research, by analyzing the regulation of the transcription process from DNA to RNA in aging and young cochleae, which mutually authenticated each other, we explored the different profiles of chromatin accessibility in the two groups, identified genes and TFs that possibly act in the pathogenesis of ARHL, and verified the SASEs and the genes controlled by them in an important position.

First, we evaluated hearing loss in C57BL/6 J mice by examining their ABR thresholds and observed that 12-month-old C57BL/6 J mice experienced severe hearing loss compared to 6-week-old C57BL/6 J mice. A worsening hearing threshold shift in the high-frequency spectrum (24 kHz, 32 kHz), which is the crucial characteristic of ARHL, was observed in aging mice relative to young mice. Individual differences in aging mice were observed in the low-frequency spectrum (4 kHz, 8 kHz). Then, changes in the cochlea and its functional epithelium or neuronal condition between the ARHL and normal mouse cochlea were evaluated; these were characterized by the loss of HCs, SGNs, and the atrophy of the SV, which corresponded to the sensory presbycusis, neural presbycusis, and metabolic presbycusis defined by Schuknecht et al. [49].

To reveal the epigenetic landscape in ARHL and identify TFs and their downstream genes active in ARHL, we performed ATAC-seq with RNA-seq in three pairs of normal and ARHL cochleae from mice. In this study, the number of highly accessible peaks in the control mice was much greater than that in the aging mice, showing that the chromatin of control cochlear tissue was more open than that of the ARHL cochlea. The less compact chromatin of the control cochlea leads to a relatively robust state of gene expression, contributing to the normal function of the cochlea. As revealed by ATAC-seq analysis, the ATAC-seq signals were enriched around the promoter-TSS regions of genes, in line with the findings of previous ATAC-seq studies. In addition, the majority of peaks mapped to intergenic regions and introns, while very few peaks mapped to exons and TTSs, again consistent with the findings in other ATAC-seq studies [50, 51]. In our study, exons and promoter-TSS regions accounted for larger proportions of the significantly different peaks, suggesting that the chromatin is more likely to be open and transcriptionally active, which is crucial for gene transcription and regulation. Specifically, open chromatin at promoter-TSS regions can permit the binding of transcription factors and other regulatory proteins, initiating the process of transcription. Similarly, open chromatin within exons, the parts of genes that encode protein sequences, could facilitate the process of transcription and the subsequent production of protein-coding mRNA. The correlation between the intensity of chromatin accessibility peaks and the expression levels of nearby genes in two distinct groups, as well as the analysis of the numbers of peaks and genes in promoter regions, in subsequent conjoint analysis corroborated this view. This suggests that changes in chromatin accessibility affecting the proportion of open chromatin in these crucial regions may have a significant impact on gene expression levels and regulatory patterns. Thus, understanding these mechanisms can provide valuable insights into cellular function and the regulation of gene expression in the progression of ARHL.

Thus, we now know that the genetic information of control cochlea was actively expressed, or a high proportion of the cells in control cochlea are transcriptionally active. The genes in close proximity to these increased peaks are involved in ‘sensory perception of sound’ and ‘‘regulation of transcription,’ which suggests that highly accessible peaks might be important for maintaining functions and activities in HCs. The potential roles of the other identified biological processes, including ‘intestinal immune network for IgA production,’ ‘NF-κB signaling pathway,’ and ‘PI3K-Akt signaling pathway,’ in cochlear function are supported by some recent research. Kensuke Uraguchi et al. found that the two NF-κB-interacting inflammatory molecules TNFα and PTGS2 were ubiquitously immunolocalized in cochlear structures in histological sections of aged cochleae [52, 53]. The nearby genes associated with the downregulated peaks were involved in ‘negative regulation of transcription’ and ‘cilium movement,’ which again served to emphasize that auditory reception cells and HCs are necessary for maintaining hearing function.

Motif enrichment analysis and motif-TF-gene prediction can provide valuable insights into molecular mechanisms. Here, several candidate genes and TFs were identified through motif analysis of the DNA sequences of different peaks. CTCF and SP1 are well-known and extensively studied transcription factors involved in various gene regulatory processes in aging. Notably, CTCF plays pivotal roles in encoding and organizing the 3D architecture of the genome with SEs, thus controlling gene expression. The high frequency of CTCF binding to its motif in this research (as the most frequent interaction observed) guided the focus of our subsequent experiments toward the identification of SEs. TBR1, EKLF, KLF6, BORIS, SP5, KLF10, NFIL3, and KLF1 also have known roles in transcriptional regulation, with each playing unique functions in aging contexts. Further studies are needed to explore the specific roles and mechanisms of these transcription factors in the process of ARHL. By analyzing this network, we also identified key TFs that may play a central role in regulating gene expression in the context of ARHL. Several known genes related to ARHL or senescence, including the forkhead gene family (comprising a diverse group of "winged-helix" proteins, e.g., Foxp1, Foxo1, Foxj1, Foxc1, and Foxg1) and the Adamts2, Arih2 (ariadne RBR E3 ubiquitin protein ligase 2), ACE2, Col13a1, and CDKN1C proteins, could be found among the genes predicted by motif-TF analysis (Fig. 3C and Table S8) [54–59]. In particular, the regulatory function of Foxg1 through the autophagy pathway during HC degeneration in presbycusis has been demonstrated in a previous study, and FOXG1 is considered a new molecular target for the treatment of age-related hearing loss [21]. Changes in footprints and insertion-site probability can provide valuable insights into the dynamics of protein‒DNA interactions and potential alterations in the regulatory landscape associated with aging. Our results show that all 10 TFs exhibit significant decreases in their TF footprints from control to aging cochlea, and the extent of TBR1, KLF10, KLF6, NFIL3, SP5, and KLF1 downregulation was greater than that for the other four TFs, which might indicate that these six TFs play a more crucial role in the identity transformation of ARHL development by binding to regulatory elements.

Using our RNA-seq data, potential TF prediction was performed. TFs from the homeobox family (Pitx1, Phox2b, and Meox1) accounted for the greatest proportion of the predicted TFs, indicating the importance of gene promoter binding events involving this TF family for preventing or promoting ARHL development. The analysis of footprint changes of the top 5 TFs reinforces this point. Notably, Foxj1 and Foxa1, as TFs from the Forkhead family, were significantly decreased, revealing the consistency of the two predictions to some extent and the crucial role of this TF family in both transcriptional regulation and organ function during ARHL progression. Notably, these TFs represent regulatory networks predicted based on computational analysis. Experimental validation and further functional studies are needed to confirm the actual regulatory relationships and elucidate the precise roles of the identified TFs in ARHL. The conjoint Venn diagram and four-quadrant analysis identified the significantly differentially expressed genes, and the expression of the top genes in the four quadrants was validated in aging mimic HEI-OC1 cells. These results could provide a better understanding of the regulatory mechanisms in ARHL and reflect the general effectiveness of the experimental approach.

Notably, in our study, the genes annotated to different peaks of the two groups did not accord with the RNA-seq results; that is, only a small number of peaks were mapped to differentially expressed genes. This is consistent with findings from other studies and supports the view that the regulation of gene expression is a highly nuanced process that relies on a multitude of functional elements and regulatory regions spread throughout the genome, some of which could be located at significant distances from the actual gene locus [51]. These distant regulatory elements, known as enhancers, could impact gene activation by facilitating the recruitment of transcription factors and RNA polymerase to the gene promoter region through the dynamic spatial organization of the genome, despite being located kilo- to mega-bases away from the gene on the chromosome.

As mentioned above, active enhancer sequences are usually located in NDRs, and our nucleosome occupancy analysis validated that the read density of NDRs in young cochlear chromosomes was higher than that in aging chromosomes. Moreover, H3K27ac is characteristic of active enhancers, and the levels of H3K27ac were decreased in aging cochleae compared to control cochleae. CTCF is a component of chromatin loops, restricting the contact of SEs and promoters, and was also identified as the top motif in the current study. These results all emphasize the essential role of enhancers in cochlear aging. The unique characteristics of SEs (including their large size, high H3K27ac density, and capacity to drive robust gene expression) make them distinguishable from typical enhancers and allow them to play more essential roles in regulating cellular processes, development, and disease, making them attractive targets for potential more effective measures for therapeutic interventions and future research. The SASEs we identified as related to ARHL showed remarkable conservation, indicating their potential roles in controlling the progression of ARHL. In aging HCs, the genes controlled by predicted SASEs were almost all obviously decreased compared with those in control cells, which reflected that the SASEs were inactive during the aging process. Blocking these conserved SEs through JQ-1 severely impaired HC viability, as did treatment with D-gal, and 8 SASE genes declined after JQ-1 treatment, revealing the critical roles that these SEs play in the maintenance of normal hearing levels. Intervening in the binding of high chromatin accessibility sites and super-enhancers with transcription factors may be a potential approach for treating ARHL in the future. By manipulating the chromatin accessibility at specific sites and regulating the binding of SEs with TFs, it may be possible to modulate gene expression and regulate the function of genes and signaling pathways related to ARHL.

Overall, this study contributes in four main ways: 1) the establishment of an ARHL mouse model and an aging mimic HC model, with a comprehensive identification of various senescence phenotypes; 2) the identification of genome-wide open chromatin regions and transcriptome profiles in the cochleae of ARHL mice; 3) the uncovering of a motif-TF-gene regulatory network and identification of key transcription factors that may play a significant role in ARHL development, through the use of individual and synergistic analyses; 4) the first identification of ARHL-associated SEs and the validation of the genes they control. Our study provides multiple insights into the similarities and differences in chromatin accessibility during ARHL development and uncovers potentially associated transcriptional regulatory mechanisms. This comprehensive analysis, as well as the identification of ARHL-associated SEs and SE-linked genes, may contribute to the development of a conservative and original therapeutic strategy targeting epigenetic dysregulation. However, it is important to note that this approach is still in the early stages of research and development. Further studies and clinical trials are needed to fully understand the effectiveness and safety of targeting chromatin accessibility and SE-TF interactions as a therapeutic strategy for ARHL. Promising advancements in the field of personalized medicine and gene therapy offer hope for the future of treating ARHL using innovative approaches like this.

### Limitations

We cannot exclude the possible contribution of epigenetic information loss in other cells, such as SGCs, to the failure of the auditory system in aging mice. In addition, the modulation of other regulatory elements, such as silencers, insulators, and locus control regions, enables these distant elements to interact with each other in three-dimensional space, and all of these elements contribute to the precise and specific control of gene activation benefiting hearing. Further understanding how all of these various regulatory regions contribute to the modulation of gene expression in different functional cells is crucial for gaining a comprehensive understanding of cell behavior and response in the context of the aging cochlea.

### Conclusion

Epigenetic factors and inactive ARHL-linked SEs play a role in ARHL. Atp6v1a, Dcun1d3, Mitf1, Sdcbp, etc., and related SASEs may be involved in the pathogenesis of HC aging. Combined analysis of chromatin accessibility and transcriptomic data revealed obviously decreased peaks at gene promoters, indicating the importance of chromatin accessibility in gene promoters for the maintenance of normal cochlear function. These findings may help identify potential targets or avenues for further research in ARHL and offer opportunities for developing therapeutic interventions to mitigate the effects of hearing loss.

### Supplementary Information

Additional file1 (ZIP 8650 kb)

Additional file2 (ZIP 18150 kb)

## Data Availability

All data generated in this study are available within the article; supplementary data will be made available on request.
