# Supplementary figures and images for: The chromatin accessibility and transcriptomic landscape of the aging mice cochlea and the identification of potential functional super-enhancers in age-related hearing loss
Source: Clin Epigenetics. 2024 Jul 4;16:86. doi: 10.1186/s13148-024-01702-1 (PMC11225416; doi:10.1186/s13148-024-01702-1)

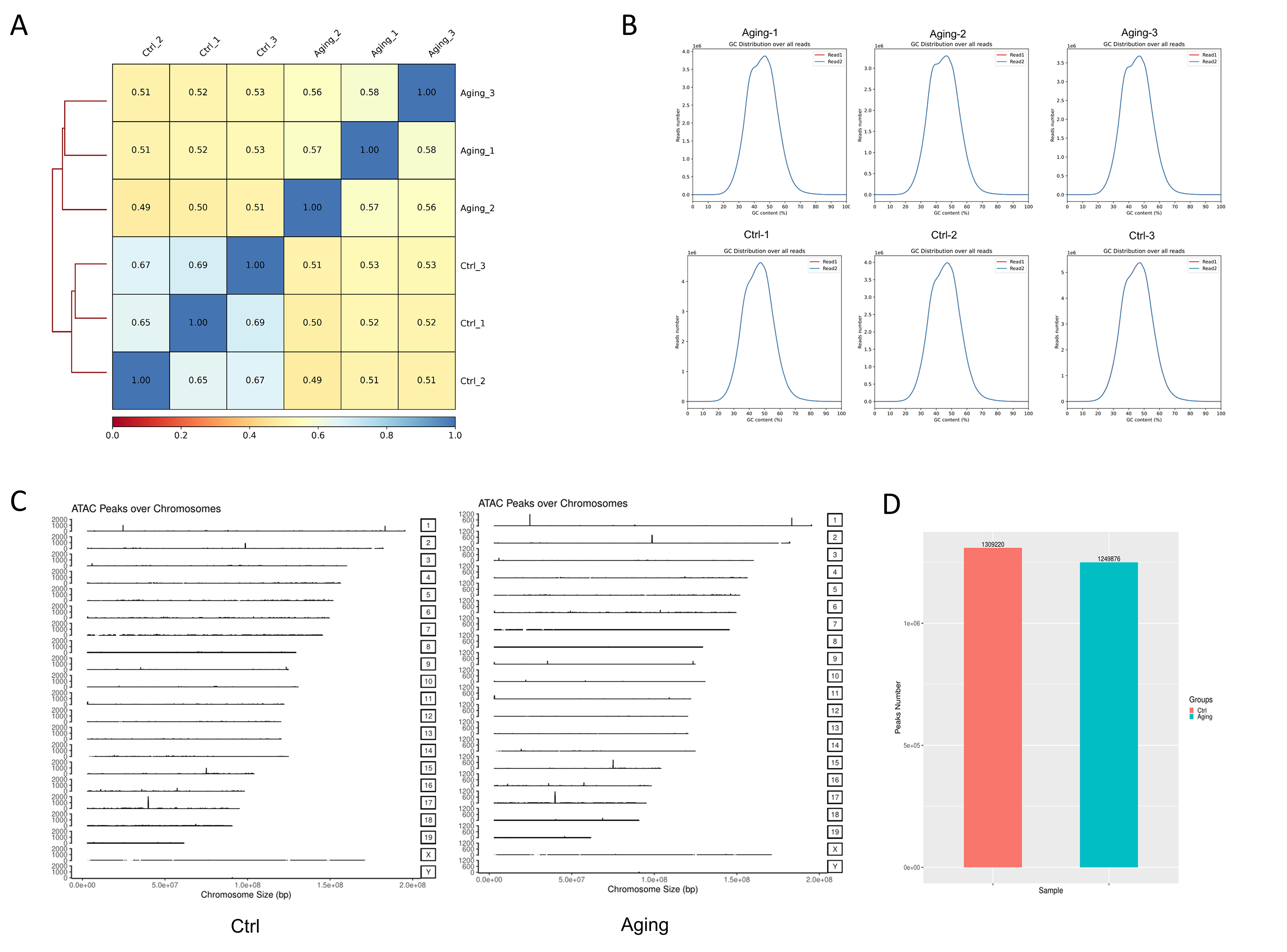

Supplement: Supplementary file 2 — Additional file2 (ZIP 18150 kb) [file 13148_2024_1702_MOESM2_ESM.zip › Supplementary figures/Fig.S1.tif]

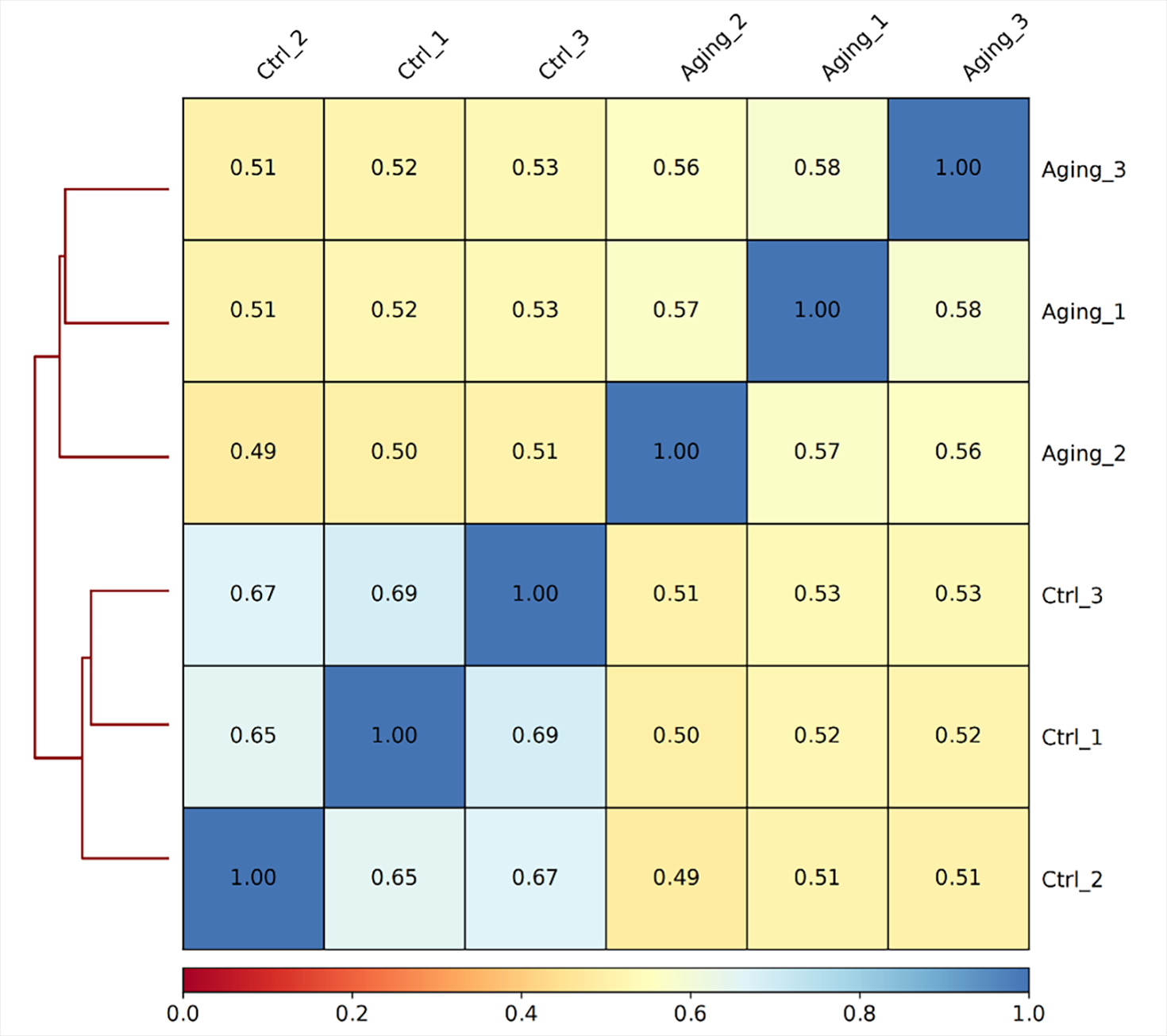

Supplement: Supplementary file 2 — Additional file2 (ZIP 18150 kb) [file 13148_2024_1702_MOESM2_ESM.zip › Supplementary figures/Fig.S1A.tif]

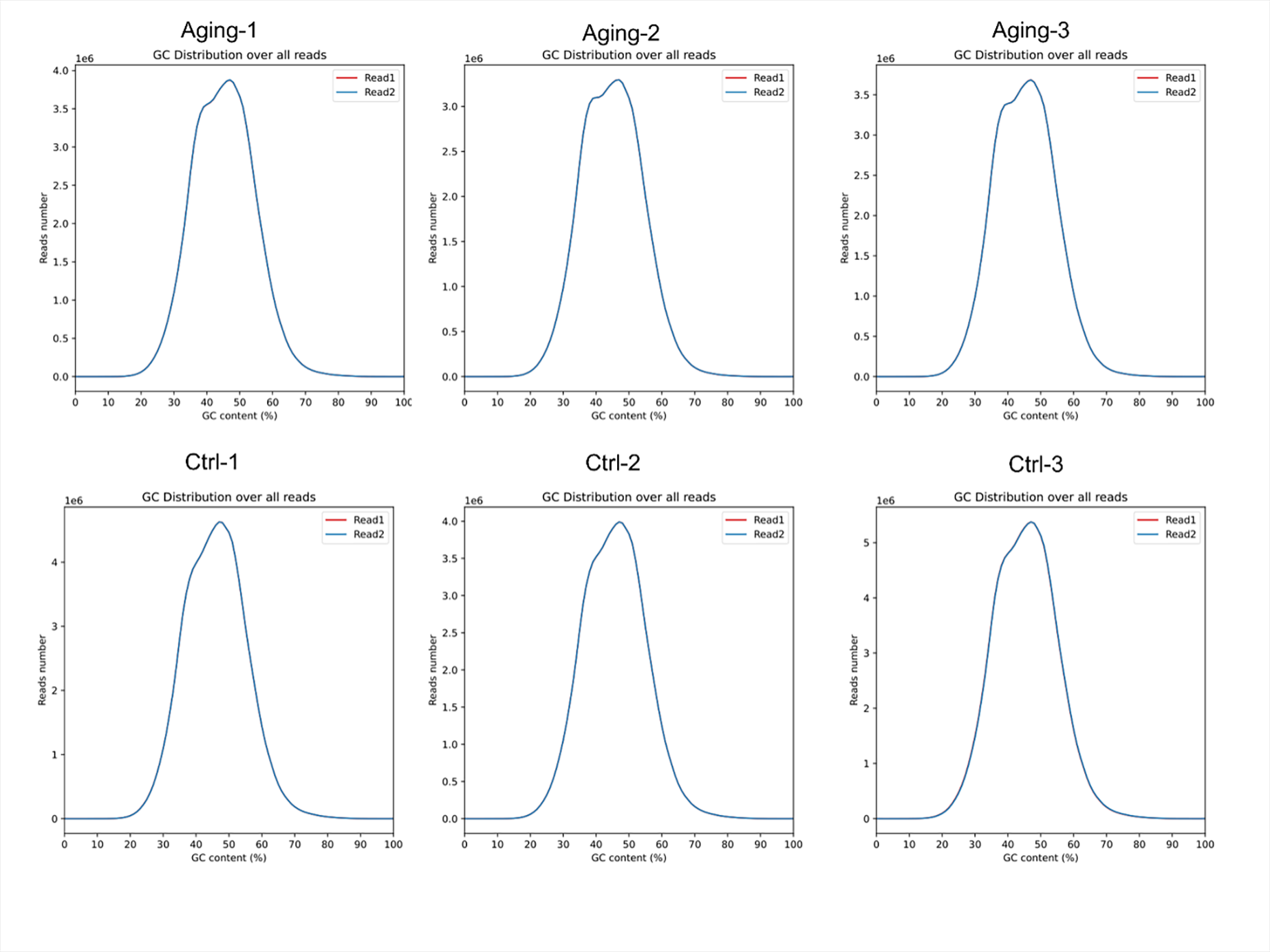

Supplement: Supplementary file 2 — Additional file2 (ZIP 18150 kb) [file 13148_2024_1702_MOESM2_ESM.zip › Supplementary figures/Fig.S1B.tif]

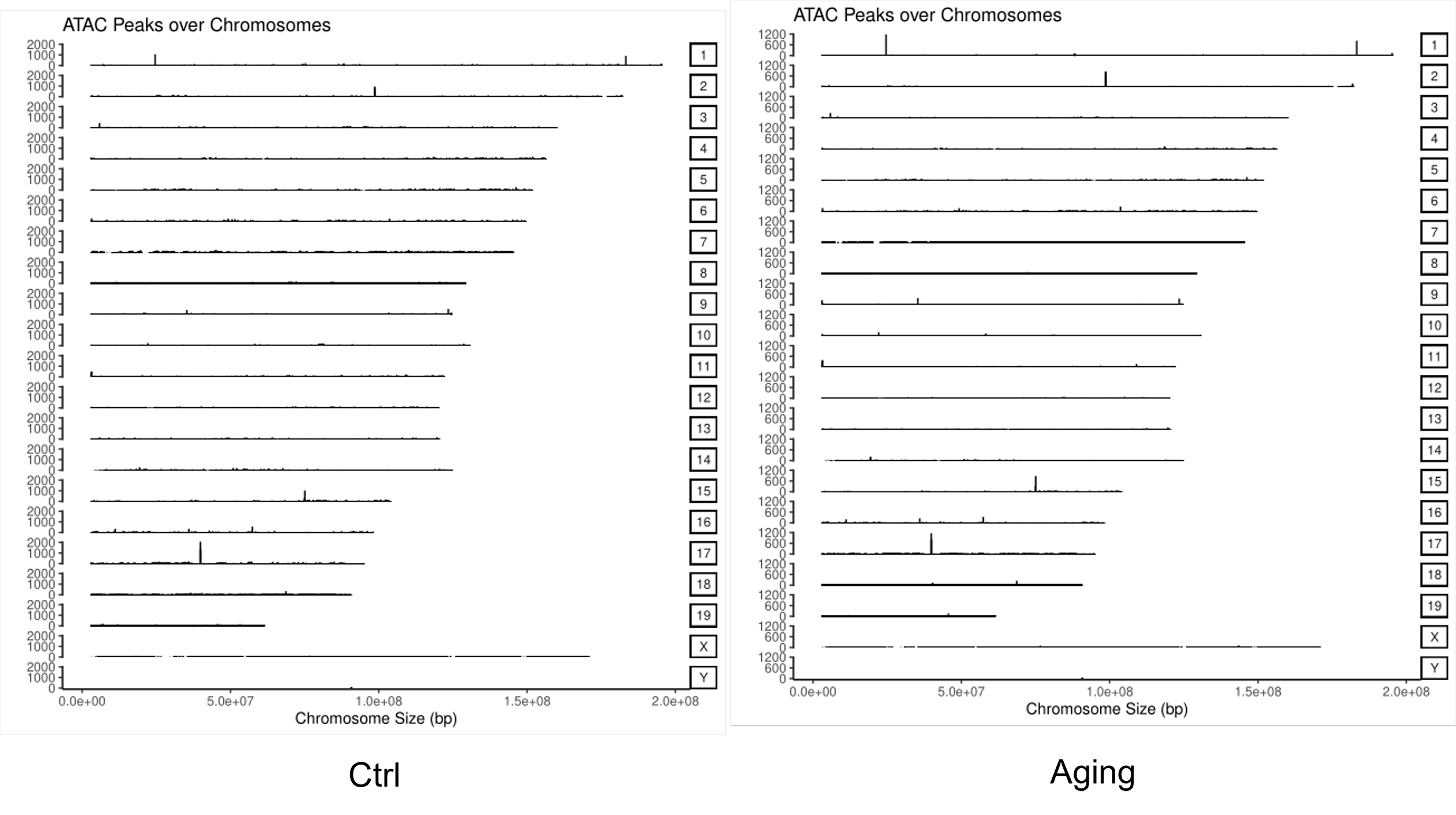

Supplement: Supplementary file 2 — Additional file2 (ZIP 18150 kb) [file 13148_2024_1702_MOESM2_ESM.zip › Supplementary figures/Fig.S1C.tif]

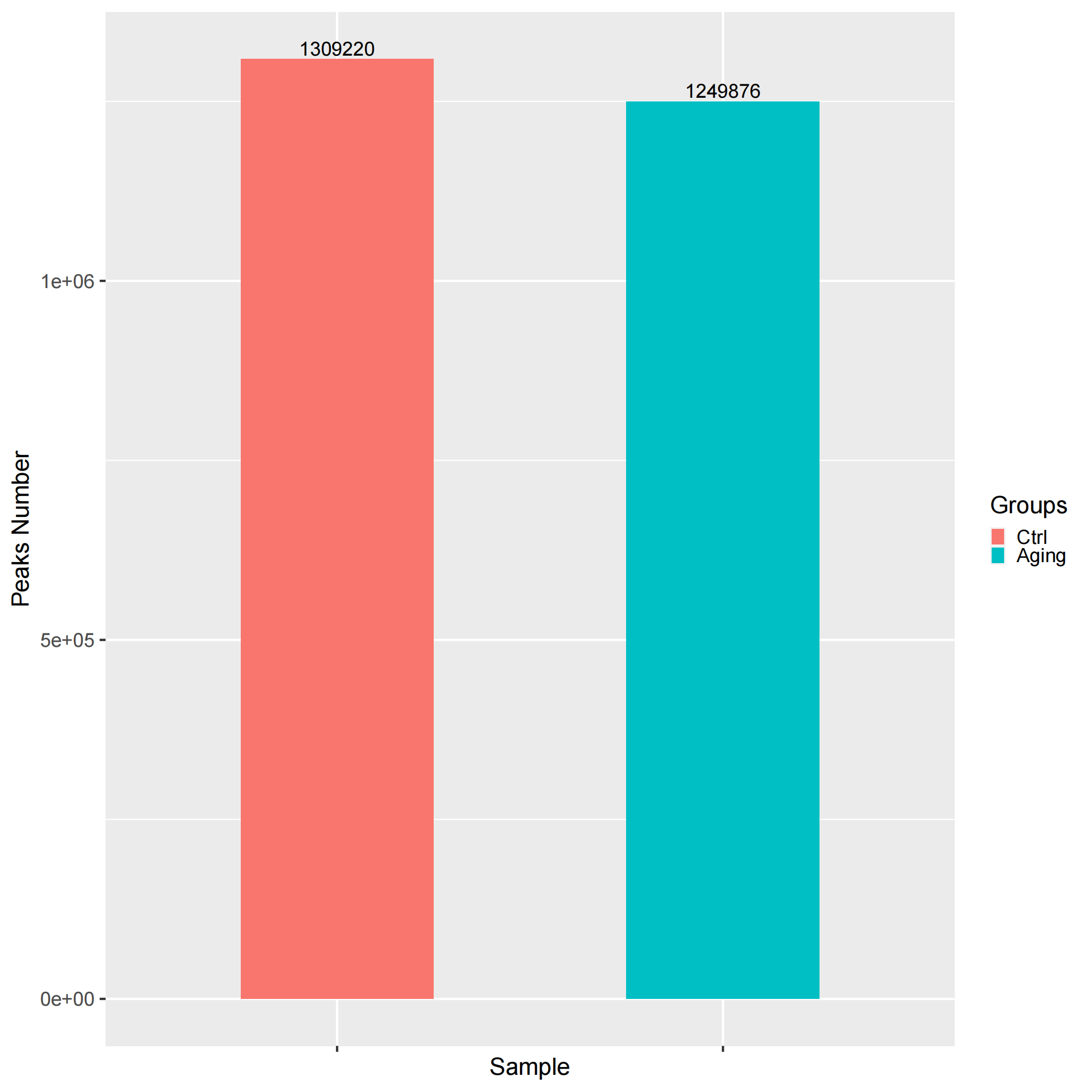

Supplement: Supplementary file 2 — Additional file2 (ZIP 18150 kb) [file 13148_2024_1702_MOESM2_ESM.zip › Supplementary figures/Fig.S1D.tif]

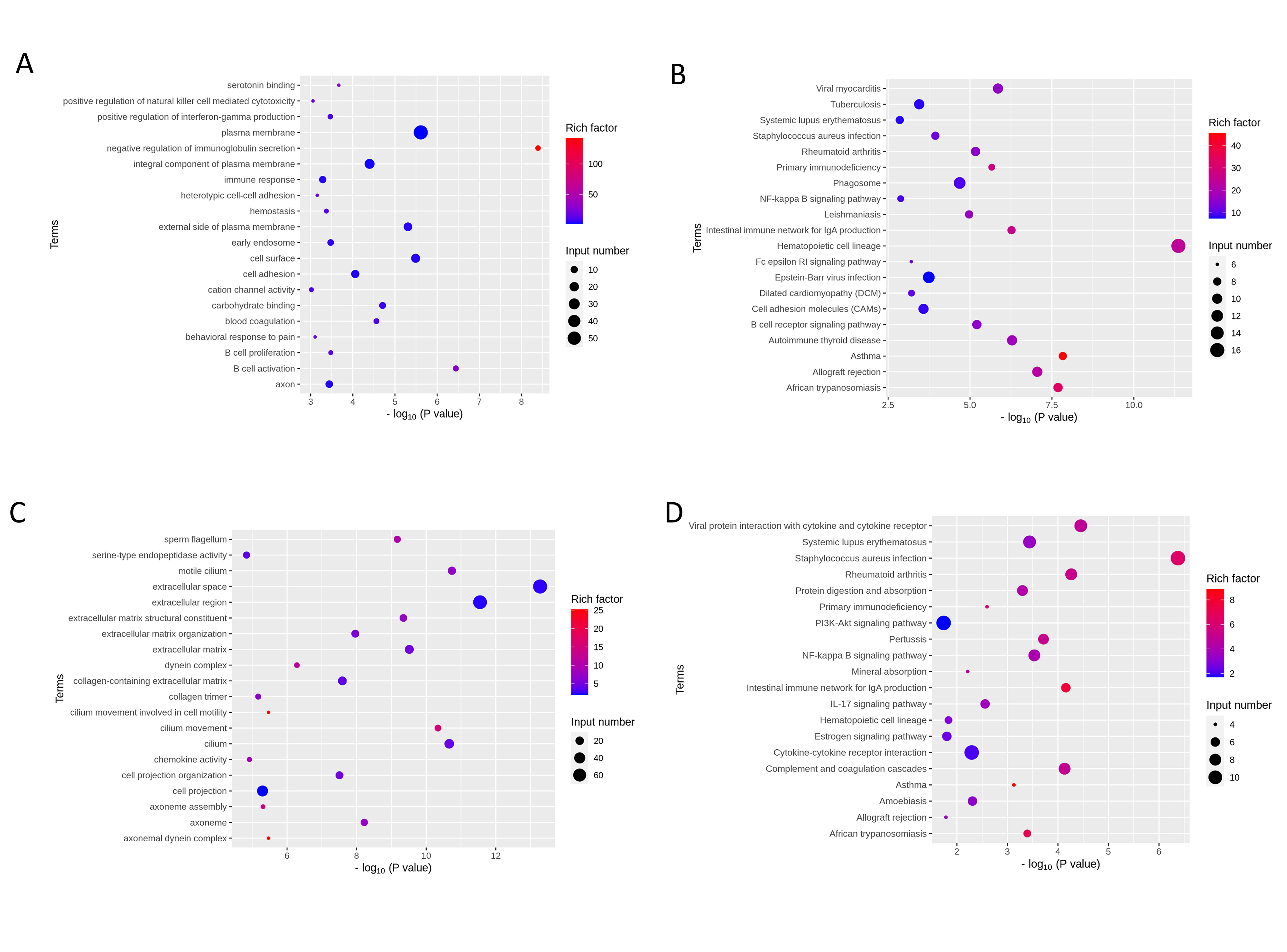

Supplement: Supplementary file 2 — Additional file2 (ZIP 18150 kb) [file 13148_2024_1702_MOESM2_ESM.zip › Supplementary figures/Fig.S2.tif]

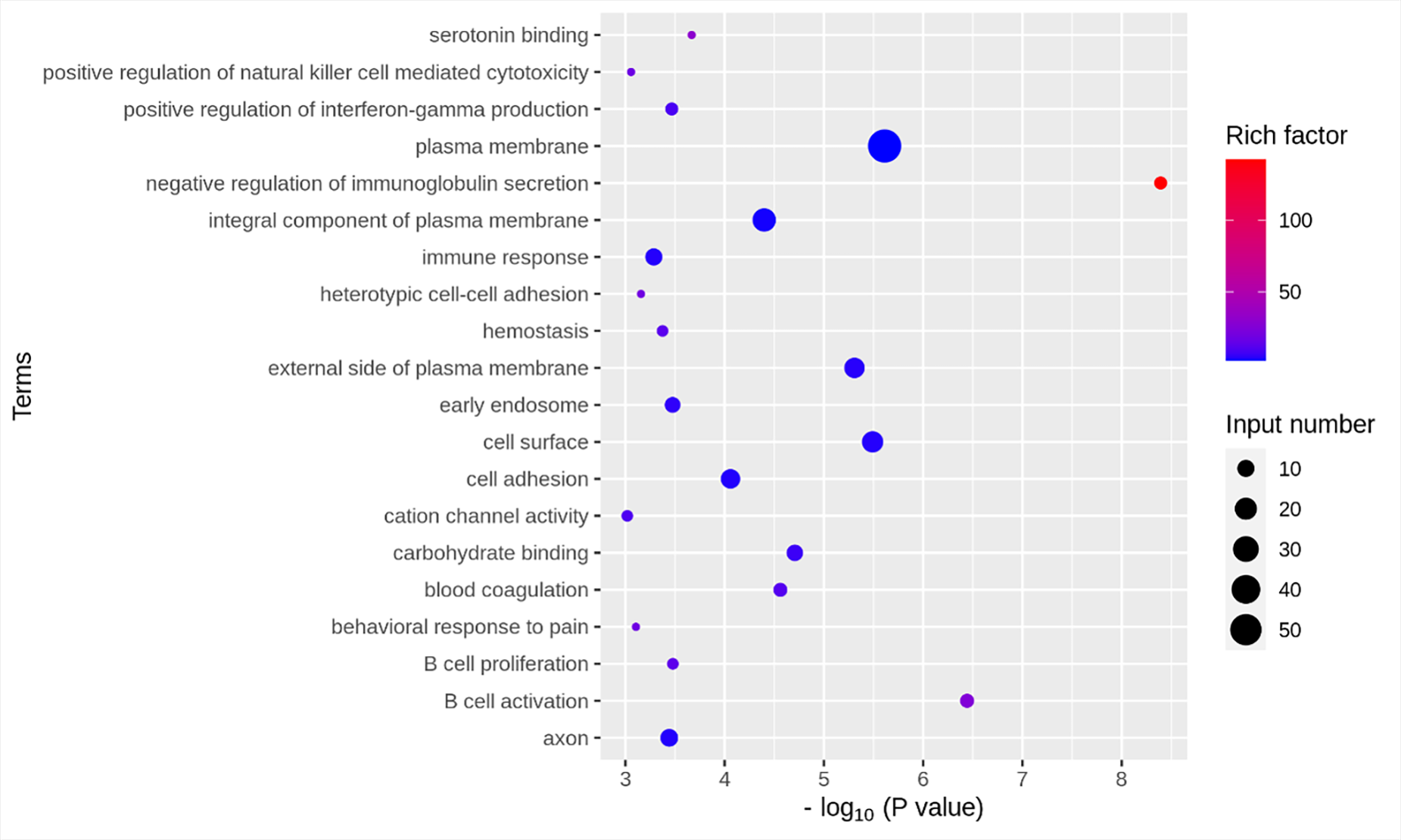

Supplement: Supplementary file 2 — Additional file2 (ZIP 18150 kb) [file 13148_2024_1702_MOESM2_ESM.zip › Supplementary figures/Fig.S2A.tif]

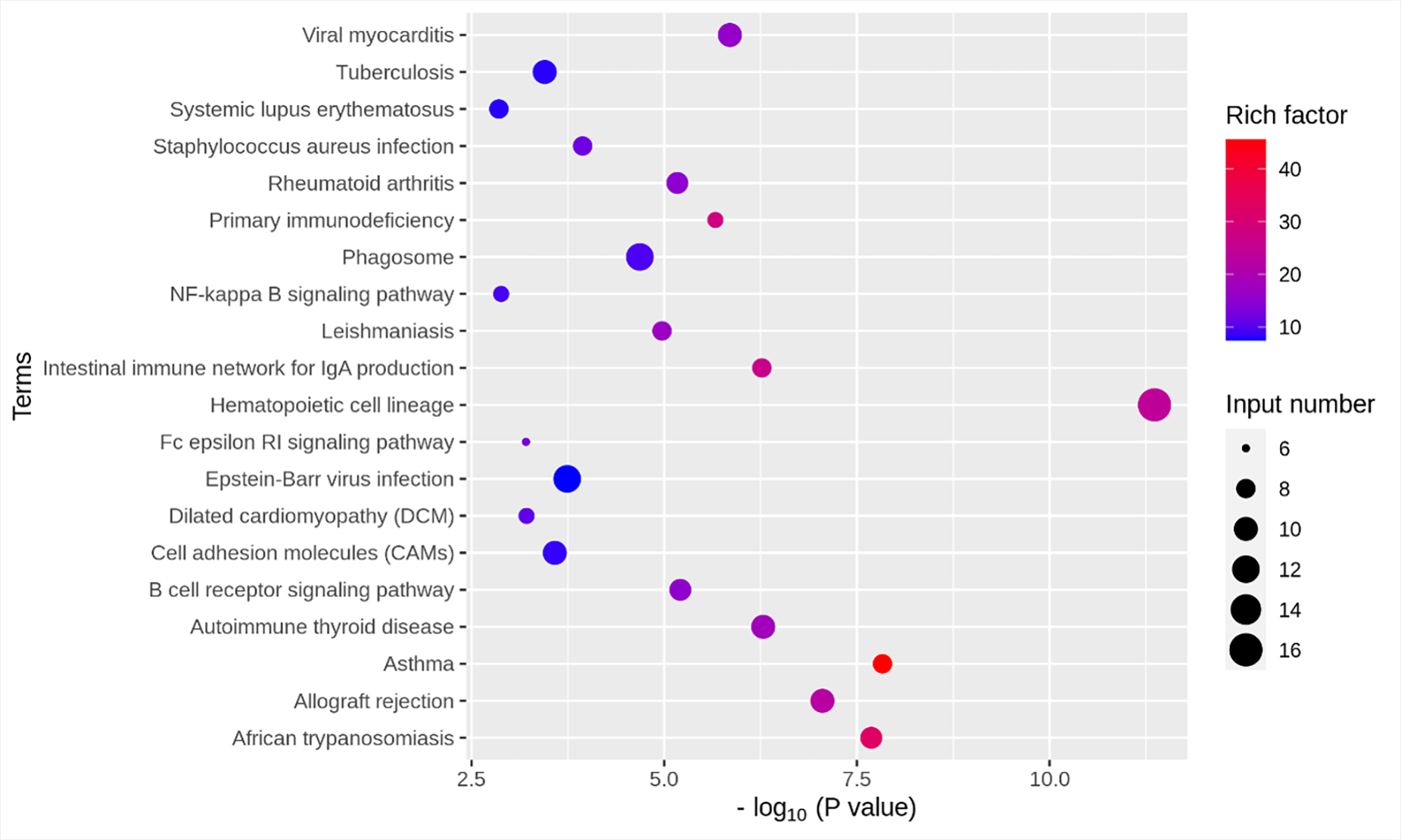

Supplement: Supplementary file 2 — Additional file2 (ZIP 18150 kb) [file 13148_2024_1702_MOESM2_ESM.zip › Supplementary figures/Fig.S2B.tif]

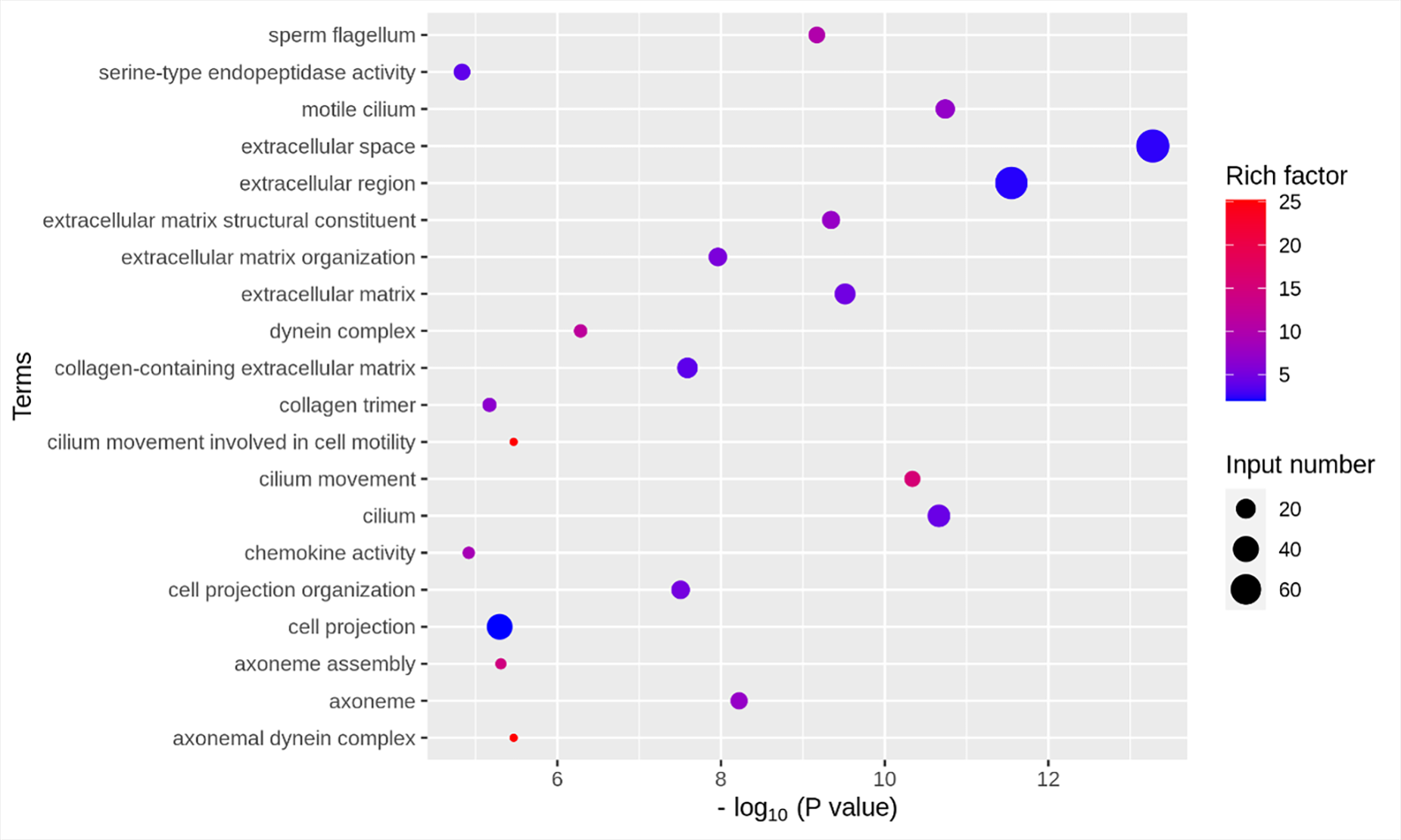

Supplement: Supplementary file 2 — Additional file2 (ZIP 18150 kb) [file 13148_2024_1702_MOESM2_ESM.zip › Supplementary figures/Fig.S2C.tif]

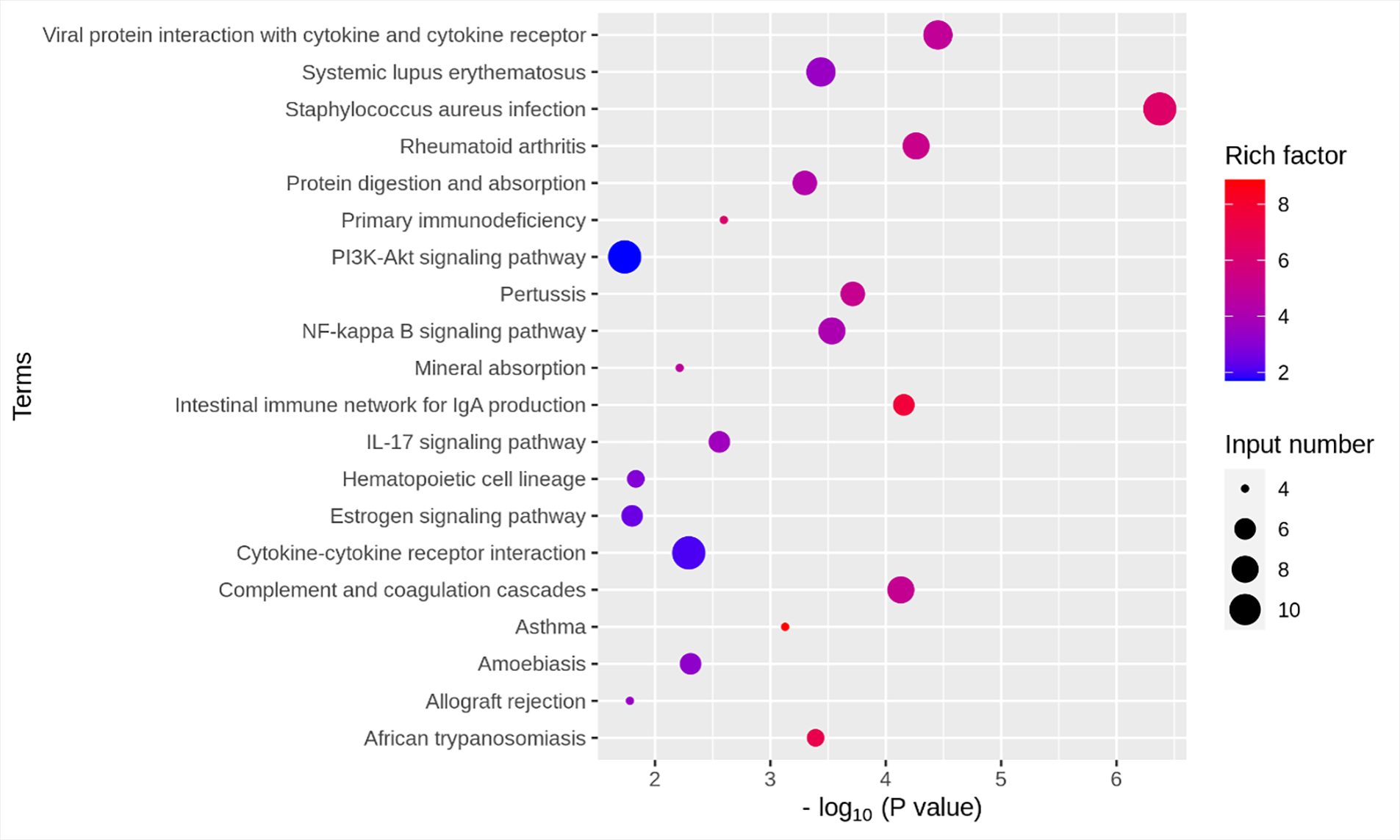

Supplement: Supplementary file 2 — Additional file2 (ZIP 18150 kb) [file 13148_2024_1702_MOESM2_ESM.zip › Supplementary figures/Fig.S2D.tif]

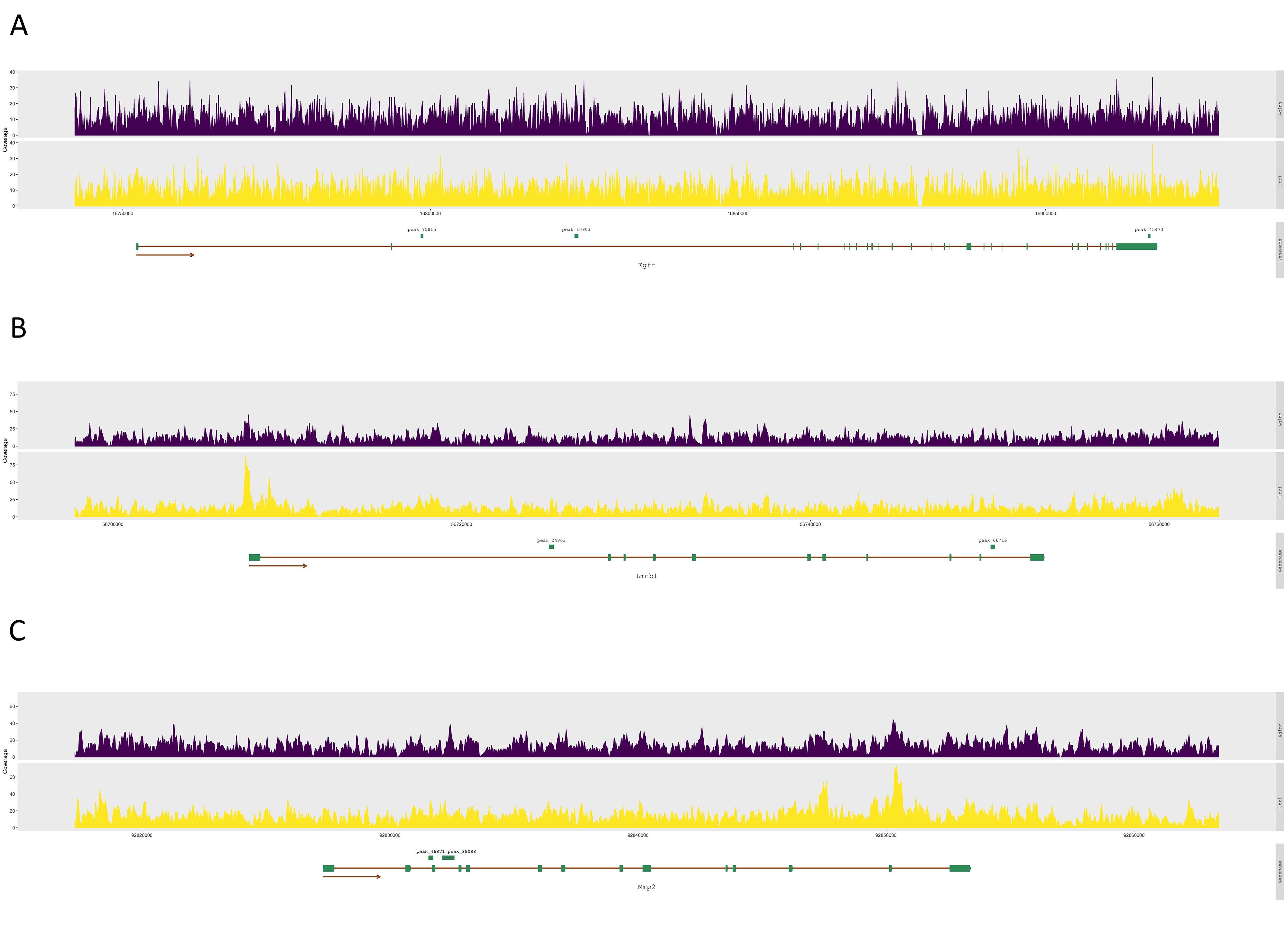

Supplement: Supplementary file 2 — Additional file2 (ZIP 18150 kb) [file 13148_2024_1702_MOESM2_ESM.zip › Supplementary figures/Fig.S3.tif]

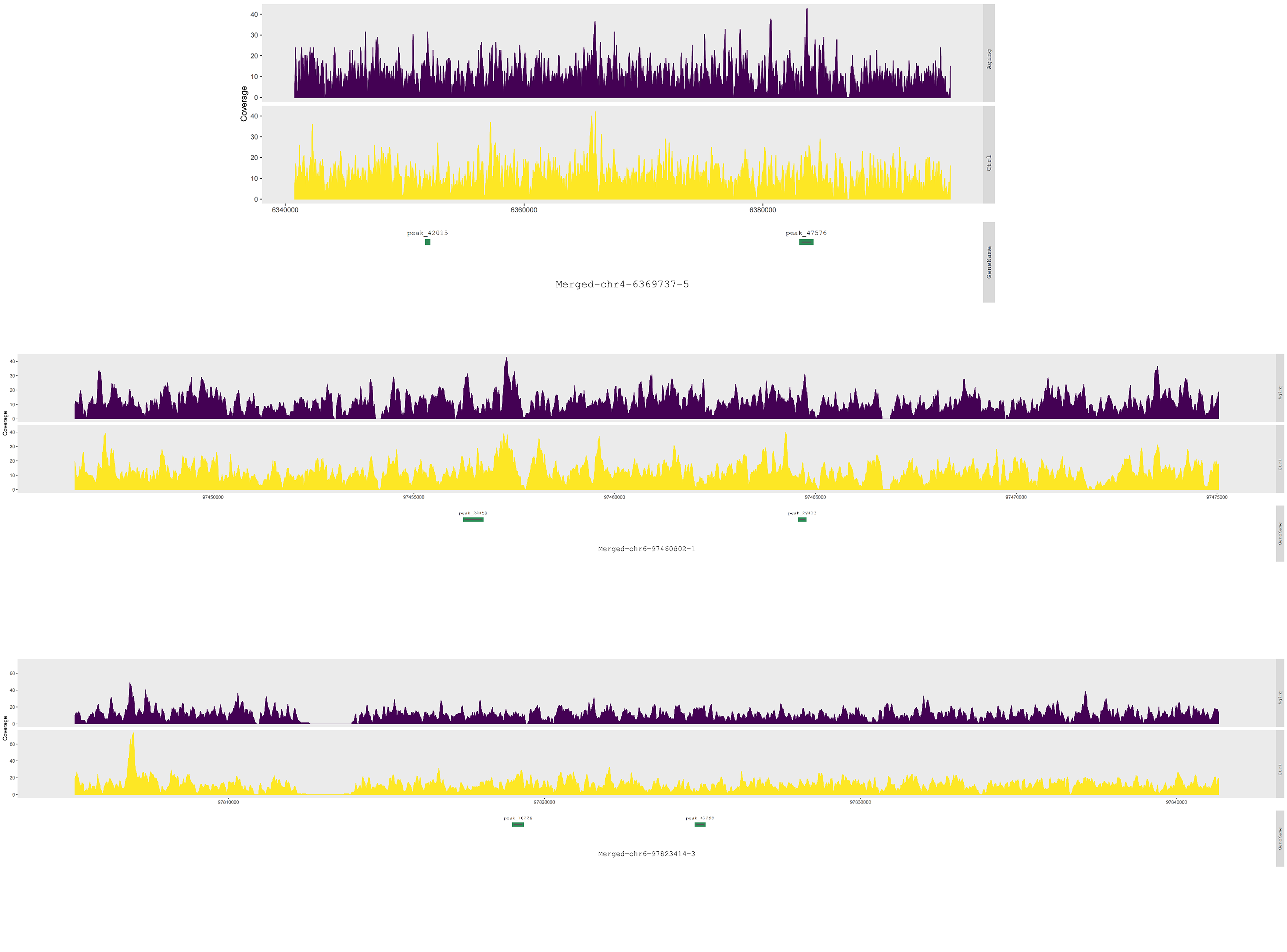

Supplement: Supplementary file 2 — Additional file2 (ZIP 18150 kb) [file 13148_2024_1702_MOESM2_ESM.zip › Supplementary figures/Fig.S5.tif]

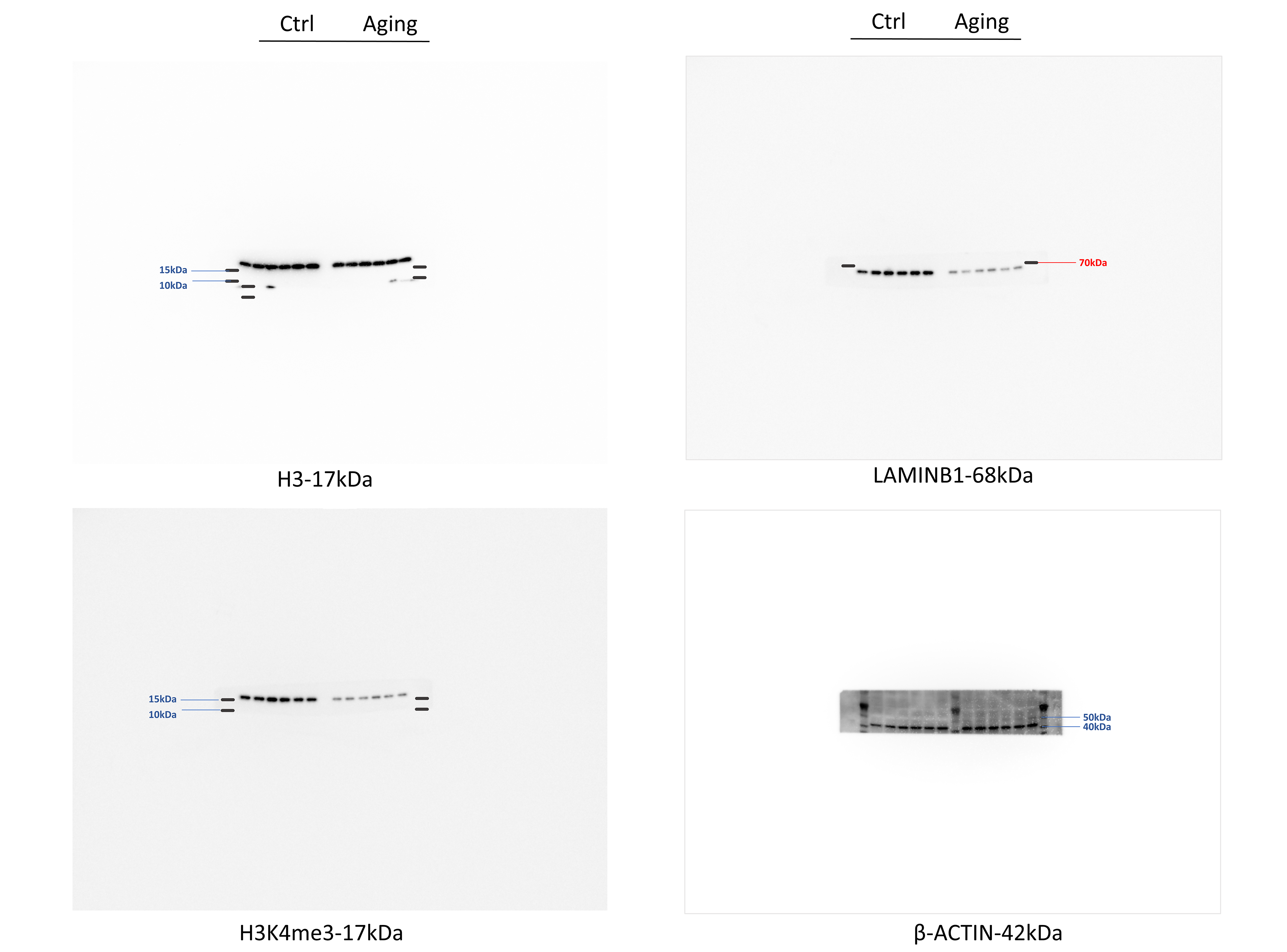

Supplement: Supplementary file 2 — Additional file2 (ZIP 18150 kb) [file 13148_2024_1702_MOESM2_ESM.zip › Supplementary figures/Fig.S6.tif]

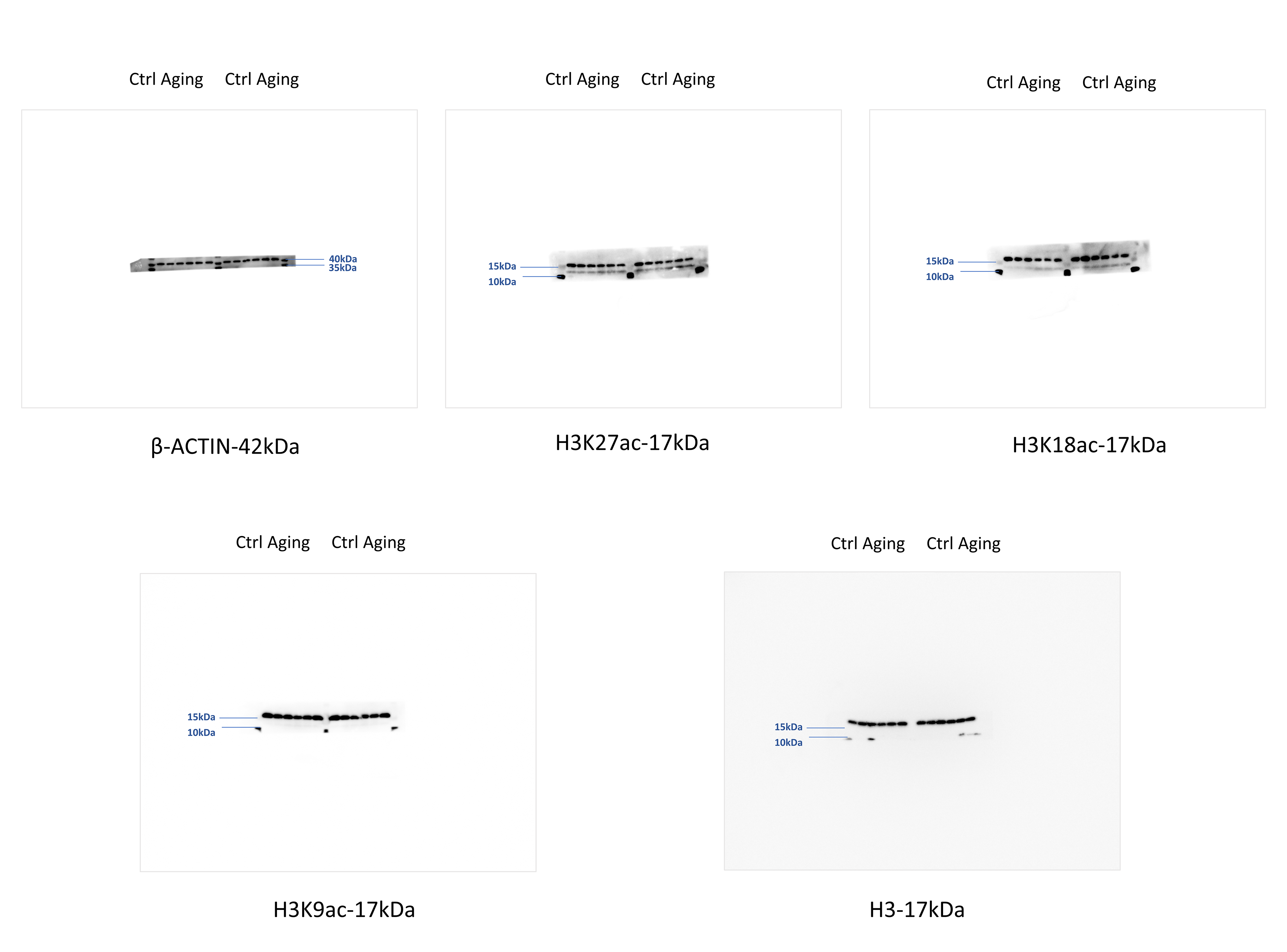

Supplement: Supplementary file 2 — Additional file2 (ZIP 18150 kb) [file 13148_2024_1702_MOESM2_ESM.zip › Supplementary figures/Fig.S7.tif]

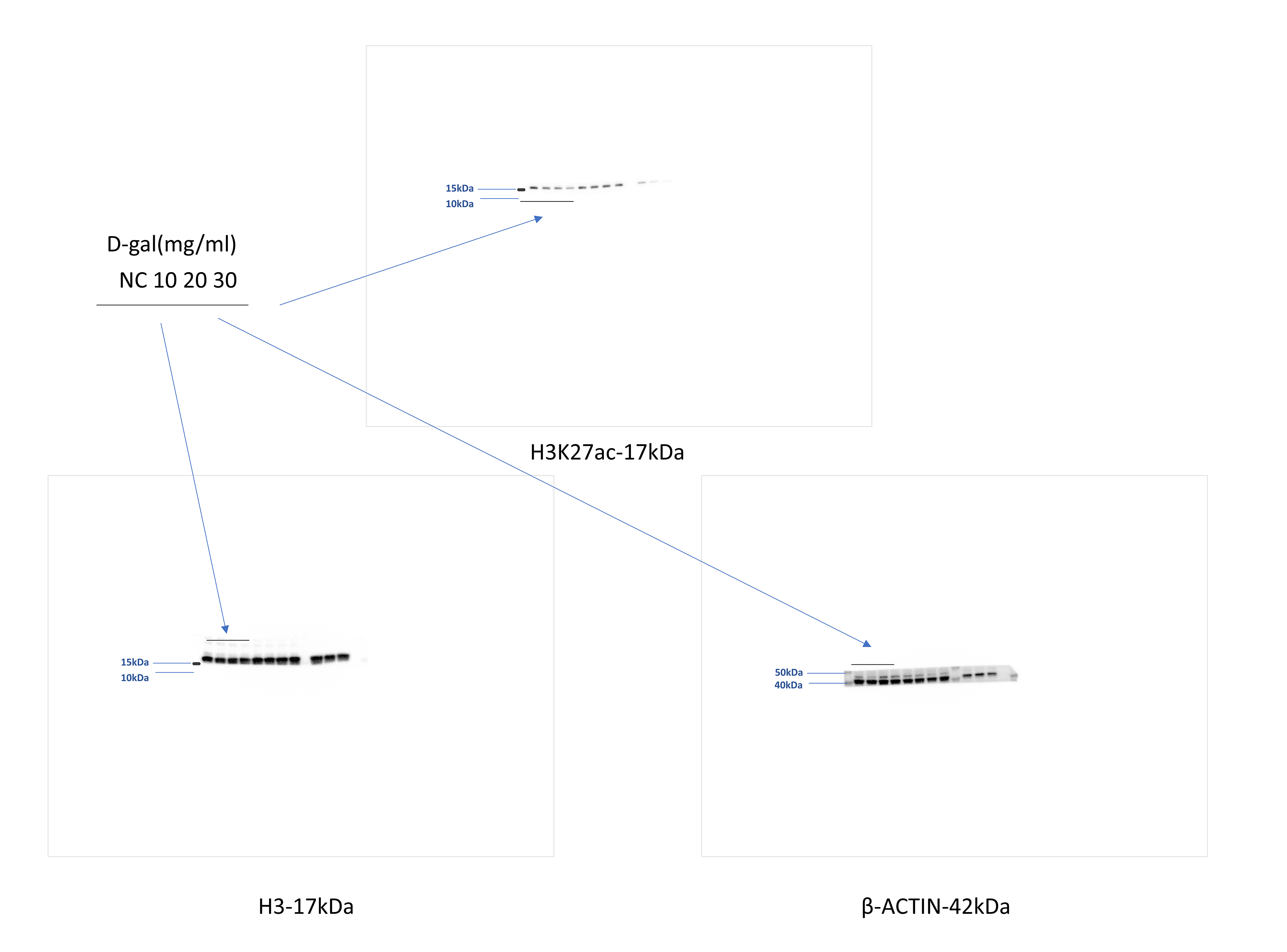

Supplement: Supplementary file 2 — Additional file2 (ZIP 18150 kb) [file 13148_2024_1702_MOESM2_ESM.zip › Supplementary figures/Fig.S8.tif]
